# A Large Scale Test of the Effect of Social Class on Prosocial Behavior

**DOI:** 10.1371/journal.pone.0133193

**Published:** 2015-07-20

**Authors:** Martin Korndörfer, Boris Egloff, Stefan C. Schmukle

**Affiliations:** 1 Department of Psychology, University of Leipzig, Leipzig, Germany; 2 Department of Psychology, Johannes Gutenberg University of Mainz, Mainz, Germany; University of the Basque Country, SPAIN

## Abstract

Does being from a higher social class lead a person to engage in more or less prosocial behavior? Psychological research has recently provided support for a negative effect of social class on prosocial behavior. However, research outside the field of psychology has mainly found evidence for positive or u-shaped relations. In the present research, we therefore thoroughly examined the effect of social class on prosocial behavior. Moreover, we analyzed whether this effect was moderated by the kind of observed prosocial behavior, the observed country, and the measure of social class. Across eight studies with large and representative international samples, we predominantly found positive effects of social class on prosociality: Higher class individuals were more likely to make a charitable donation and contribute a higher percentage of their family income to charity (32,090 ≥ *N* ≥ 3,957; Studies 1–3), were more likely to volunteer (37,136 ≥*N* ≥ 3,964; Studies 4–6), were more helpful (*N* = 3,902; Study 7), and were more trusting and trustworthy in an economic game when interacting with a stranger (*N* = 1,421; Study 8) than lower social class individuals. Although the effects of social class varied somewhat across the kinds of prosocial behavior, countries, and measures of social class, under *no* condition did we find the negative effect that would have been expected on the basis of previous results reported in the psychological literature. Possible explanations for this divergence and implications are discussed.

## Introduction

Only a few themes in personality and social psychology have attracted interest from both the expert audience and the general public to a similar degree. One of these themes is certainly the effect of social class on prosociality. Recent social psychological research has presented evidence of a negative effect of social class on several prosocial behaviors [1–3]. In these studies, higher class individuals were found to be less charitable, less trusting, less generous, and less helpful than lower social class individuals. These findings have been implemented in a social-cognitive perspective on social class [4], have been used as a paragon for a newly developed psychological decision-making process of prosociality [5], and have been eagerly picked up by the lay press [6,7].

However, there are important reasons to question the proposed negative relation. On the one hand, research outside the field of psychology has not been in line with this psychological perspective [8–11]. On the other hand, some methodological weaknesses in this psychological research lower its generalizability and conclusiveness. In the present research, we therefore thoroughly examined the effects of social class on prosocial behavior by using a variety of large representative panel studies. By doing so, we were additionally able to check for the moderating role of some potential factors of influence (kind of observed prosocial behavior, observed country, and measure of social class) and to make more generalizable statements [12,13].

### The Proposed Negative Effect of Social Class on Prosocial Behavior

In the psychological literature [1,2,4,14] it is contended that lower social class individuals should show enhanced prosociality for the following reasons. Lower social class individuals live in more stressful and threatening environments than higher class individuals [15], and they are also more vigilant to those threats [16]. In addition, they lack economic independence due to their small economic resources [17,18]. As a result, they prioritize contextual explanations for their fates and life experiences and feel a diminished sense of control compared with higher class individuals [19–21]. Lower social class individuals realize that they have to rely on others to achieve their aims and, thus, they turn more toward their environment—they show more signs of affiliation such as headnods and posture and were less tied up with their cell phones in an induced interaction with a stranger [22], were more accurate in judging the emotions of photographed faces and actual interaction partners [14], and have been found to be generally more compassionate [23–25]. Because they are more worried about and involved in their environment, finally, they “will act in a more prosocial fashion to improve others’ welfare” ([1], p. 772).

Only a few specific scientific investigations have been conducted to examine this theoretical rational. In the most prominent one, Piff et al. [1] conducted four studies. In the first study, people from the lower social realms allocated more points to a stranger in a dictator game. In the second study, compared with higher class individuals, lower social class individuals favored donating higher proportions of their annual salary to charity. In the third study, people from the lower social classes gave more money-like points to an assigned stranger in a trust game. Last, in Study 4, lower social class individuals were more helpful to an unknown lab mate—they were more likely to sacrifice their time for this lab mate after she had been late (and had already cost time) in the first place. All in all, the empirical results consistently supported the theoretical suppositions and demonstrated the validity of the “negative effect” hypothesis. Similarly, Guinote et al. [2] showed that individuals with a lower status were more prosocial than high-status individuals. More specifically, in four studies that used status manipulations such as falsely telling participants that the prestige of their department or art school was low (vs. high) compared with other departments or art schools, lower status individuals reported more prosocial life goals and showed more helping behavior in the lab (i.e., picked up more pens that were “involuntarily” dropped by the experimenter) than individuals who were induced to feel that they had high status.

In addition, there is further evidence on the relation between social class and self-interested unethical behavior that supports the “negative effect hypothesis.” For example, across six experiments, Dubios et al. [26] showed that higher social class individuals were more likely to perform unethical, self-beneficial behaviors such as cheating when throwing dice or to report falsely keeping large amounts of change for themselves. Similar results have been found for illegal behaviors such as shoplifting [27] and cheating on taxes [28,29]. Moreover, across seven experiments, Piff et al. [30] provided evidence that higher social class individuals were more likely to take goods from others and lie in negotiations and showed higher propensities to engage in unethical behavior at work (e.g., making personal long-distance phone calls at work or overcharging customers to increase sales and earn a higher bonus).

All in all, these findings are in line with the psychological mainstream, which has identified higher class individuals as not-so-good or even bad persons [30–35]. Scholars in psychology have even gone so far as to promote a new social-cognitive theory on social class that incorporates much of the mentioned research and explains for instance why higher class people are more likely to drive their cars inconsiderately or why they are more utilitarian in a social dilemma [4]. Furthermore, and grounded on the assumption that social class represents some sort of culture, Keltner et al. [5] utilized the “negative social class effect” [1] as a prime example for a new theory on prosociality in general. According to this theory, several emotions, values, and sociocultural appraisals (e.g., norms, perceived benefits, or perceived costs of nonprosociality) influence prosocial behaviors, and because they differ between the social class realms, they promote the prosociality of lower social class individuals but hamper the prosociality of higher class individuals.

### Reasons to Question the Proposed Negative Effect

#### Research Outside the Field of Psychology

Outside the field of psychology, however, there is no consensus about the direction of the effect of social class on prosociality, and research has provided evidence for two nonnegative classes of findings.

The first class of findings supports a positive effect of social class on prosocial behavior. In a large nationwide survey in the United States, the Consumer Expenditure Survey, James and Sharpe [36] found that higher social class individuals were more likely to make any kind of charitable donation. In addition, using data from the United States Internal Revenue Service, Gittell and Tebaldi [8] demonstrated that income and education—two well-known indicators of social class—were considerable determinants of the monetary amounts of the donations. Hughes and Luksetich [9] further corroborated the positive effect of social class on prosocial behavior with data from the Center on Philanthropy Panel Study and the Panel Study on Income Dynamics. Moreover, the positive association was not limited to the United States or to the act of donating but was also found in Canada [37,38] and Taiwan [39] and for other prosocial behaviors such as volunteering [10,11,40] (for some results within the field of psychology, see [41]).

The second major group of findings posits a u-shaped relation between social class and prosocial behavior [36,42–44]. On the basis of the 1992 Survey of Giving and Volunteering in the United States, Hodgkinson et al. [42] discovered the u-shaped curve when determining the relation between the percentage of contributed household income and total household income. This trend remained in a subsequent Survey of Giving and Volunteering in the United States [40] and was recently confirmed by data from the Internal Revenue Service [45]. However, because the tail of the curve is usually higher at the lower end of the social class scale than at the higher end, the u-shaped curve has also been repeatedly presented as evidence that lower social class individuals are more prosocial than higher class individuals [1,4]. Similarly, relying on data from the American Consumer Expenditure Survey from 2007, Greve [46] reported in the public media that households from the lower income groups who made donations (mean income = $10,531) donated 4.3% of their income to charity, whereas households from the highest income group (mean income = $158,388) gave only 2.1% of their income to charity.

However, this pattern of results is likely to be caused be the single observation of donor households. It is known from other research that lower social class households are less likely to make any donation at all [36]. Thus, it is necessary to include donor and nondonor households in a joint analysis. When doing so, the u curve is likely to transform into a linearly increasing curve that indicates that higher social class households donate a greater percentage of their income to charity [43,44].

The theoretical considerations underlying research outside the field of psychology are not less persuasive than the psychological ones and, in fact, are also based on some fundamental psychological processes themselves. These include the different economic resources and accordingly, the different costs of prosociality. Individuals in lower social classes possess less money than higher social class individuals [17]. Thus, for lower social class people, there might be nothing or at least less left to give (even as a proportion of their income). On the contrary, for higher class individuals, it is easier to give because they simply have more to give. This line of reasoning becomes clearer if the prosocial act is conceptualized as a cost-benefit consideration: Because lower social class individuals suffer from resource scarcity, the subjective costs of giving something away are higher for them compared with individuals in the higher realms of social class. It is known that the likelihood of a prosocial act is reduced if the costs of the act are increased [47]. Thus, the higher costs of prosociality might undermine lower social class individuals’ inclinations to act benevolently. Trautmann et al. [41] recently discussed and partly demonstrated how the reasoning behind different cost-benefit scenarios made by different social classes might contribute to the different effects on unethical behavior.

#### Methodological Issues of Previous Work in the Field of Psychology

The key studies that posited a negative relation between social class and prosociality [1,2] were based on rather small samples (for [1]: 81<*N*<155; for [2]: 44<*N*<82; for the studies that addressed unethical behavior, the sample sizes were 90<*N*<274 [30] and 81<*N*<151 [26]; altogether, *median* = 115). Using multiple small- to medium-sized samples, such studies have been criticized in general and may be less credible than we used to think [48–51]. Whereas the use of multiple studies conveys an impression of complied replicability, robustness, and persuasiveness, in fact, statistical power (the chance of detecting effects that actually exist) decreases when a greater number of statistical tests are executed [48,49,51,52]. The statistical power might then even be so low that it prompts questions about how all of the studies could have obtained significant results.

Such a discussion has recently occurred with regard to the influence of social class on unethical behavior. In a multistudy paper using seven experiments [30], higher social class individuals were shown to be less ethical than people in the lower social realms. Francis [53] critically evaluated these results and argued that publication bias led to this unlikely pattern of results (for more on the debate surrounding these results, see [54,55]). When computing the observed power of each single study and determining the final likelihood that all seven studies would reject the null hypothesis if effects actually existed, Francis [53] found a probability of only .02.

Among others, Schimmack [51] argued that some sort of bias (e.g., sampling bias, publication bias, and/or design bias) is likely to contribute to the unlikely pattern of many significant findings in a single article (see also [48–50,53,56]). And indeed, what also struck Francis [53] about the findings on social class and unethical behavior “was the consistency of the results across different definitions of social class and measurements of unethical behavior” (p. E1587). At first glance, these findings foster the generalizability of the results such that no matter how social class was assessed, increases in social class apparently enhanced unethicality. But on the other hand, the small overall statistical power indicates that the effect might not be that reliable [51,57,58].

### Possible Moderators of the Negative Effect

The previously presented arguments (research outside the field of psychology and the small overall statistical power) are at odds with the “negative effect” hypothesis or at least create the impression that previous results on effects of social class on prosocial behavior [1–3] might not be as robust as previously thought. This is why we also analyzed possible moderators that may function as boundary conditions of this effect.

#### Observed Prosocial Behavior

Prosociality describes a large variety of behaviors that benefit others [59–61]. Yet, in the nonpsychological literature that contradicted the proposition that social class negatively influences prosociality, the most dominant way of assessing prosocial behavior was via the likelihood, amount, and percentage of charitable giving [8,9,39,42,45] or volunteering [11]. The psychological literature, on the contrary, based its propositions on many different prosocial acts such as the allocation of points in various economic games [1,3,62] or helping behavior in a laboratory situation [1,2].

#### Observed Country

The United States has an only slightly elaborated social-welfare system that is difficult to compare with those of European countries (e.g., Germany, see [63]). Hence, in the US, nonprofit and religious organizations provide a great deal of support for those in need [9,64]. It is plausible that this lack of government help has enhanced solidarity among the less privileged and has led to a climate of prosociality among people in the lower social realms. Thus, culture might also act as a moderator of the effects of social class (see also [65–67]). Recent research on the effects of social class on unethical behavior fosters this assumption and gives reason to presume that the negative effect of social class on prosocial behavior might vary between countries [68]. Across seven studies with American samples, Piff et al. [30] found that higher social class individuals lie and cheat more often and are more likely to take goods from others than their counterparts in the lower social classes. By contrast, using data from a large scale representative panel, Trautmann, van de Kuilen, and Zeckhauser [41] could not find this general propensity in the Netherlands.

Moreover, Chen et al. [3] found that children from high-income Chinese families allocated fewer stickers in an adopted dictator game, whereas Benenson et al. [62] found the opposite effect among British children. As similar tendencies may apply to the effects of social class on prosocial behavior among adults, it is worthwhile to consider the observed country as a potential moderator.

#### Different Measures of Social Class

Social class is predominantly conceptualized as a composition of objective indicators of socioeconomic status (income, education, occupational prestige; [1,3,4,17,62,69–71]), and most studies outside the field of psychology employ at least one of these indicators. But recently and especially inside the field of psychology, research has proposed that social class is not limited to these objective measures of socioeconomic status but instead may also comprise individuals’ perception of social standing compared with others in society [1,26,66,69,72–74]. This alternative measure of social class is highly subjective and may indeed differ from the position one would expect on the basis of socioeconomic indicators [75] and, thus, might show different effects on prosociality.

### The Present Research

To thoroughly examine the effect of social class on prosocial behavior, we conducted eight studies. In contrast to previous studies in the psychological field using relatively small and nonrepresentative samples that, in sum, led to problems with statistical power and lowered the generalizability of their research, we used large representative panels in all of our studies. Specifically, we used the German Socio-Economic Panel (SOEP), the American General Social Survey (GSS), the American Consumer Expenditure Survey (CEX), and the International Social Survey Programme (ISSP). These surveys were professionally conducted by large research organizations that also anonymized and de-identified the data and made the data publicly available on their websites. As we only reanalyzed these publicly available data sets, a particular ethical approval for our studies was not required.

The variety of panels, on the one hand, guaranteed a realistic variation of social class and is therefore preferable to the previously used samples that sometimes consisted entirely of students (for a further discussion on the problematic use of WEIRD samples in the research on social class, see [76]). On the other hand, it enabled us to additionally test for potential moderators of the effect of social class on prosociality. First, the panels we used were not limited to a certain country such as the United States or Canada but instead covered a broad range of countries. Therefore, we were able to determine whether or not the effects of social class on prosocial behavior differed between countries. Second, the panels provided, on the one hand, objective and state-of-the-art indicators of social class (income, education, and occupational prestige) that could be combined into a composite measure of objective social class according to previous studies [69]. On the other hand, some of the investigated panels additionally assessed respondents’ subjective social class, and therefore, we were able to test for effects of different measures of social class. Third, the examined panels offered various behavioral measures of prosociality, and thus, we were able to analyze our data with regard to a moderating role of the investigated behavior. Specifically, we used data on actual donation behavior (Studies 1–3), volunteering (Studies 3–6), helping in everyday situations (Studies 7), and trust and trustworthiness in a trust game (Study 8).

Taken together, the following studies allowed us to determine the effects of social class on prosocial behavior in large representative samples and furthermore to test for a moderating role of the observed country, the measure of social class, and the assessed prosocial behavior.

## Study 1: Effect of Social Class on Donating (SOEP)

The goal of Study 1 was to examine whether social class has an influence on donation behavior in Germany. Donating is one of the best studied and most widespread acts of prosociality [77,78].

As outlined in the introduction, a special feature of the effects of social class on donating has been the repeatedly found u curve [36,40,43–45]. According to this research, people in the lower social classes donate the largest percentage of their income and even more than those in the highest social classes. The most parsimonious persons are those in the middle of the social class distribution. However, as stated before, the u curve may be caused by the methodological artifact of exclusively examining donor households.

Therefore, we tried to investigate the effects of social class on donating with two separate approaches. First, we examined whether the probability that a household would donate anything to charity would be higher or lower with elevated social class. Second, we investigated whether the percentage of household income that was contributed increased or decreased with elevated social class. Thereby, we distinguished between an analysis of only donor households and an analysis that integrated all households. Like previous research [36,40,44,46], we analyzed donation behavior at the household level because donations are often made by both partners together.

### Method

#### Participants

The data used in this study were provided by the German SOEP (Version 29) of the German Institute for Economic Research. The SOEP is a large longitudinal survey of private households and persons in Germany started in 1984 (see [79], for details). Due to the high stability of the sample (about 94% in consecutive years) and the inclusion of new participants, the sample contained 22,870 individuals and 10,745 households in the year 2010. The donations from each household for the year 2009 were gathered in the year 2010. A total of 1,382 households were excluded from our analyses because none of the household members answered the donation questions or the indicators of social class. The remaining 9,363 households ranged in size from 1 to 14 persons (*M* = 2.28, *SD* = 1.18), and the mean age of all household members was 42.66 years (*SD* = 22.23).

#### Objective social class

We computed a composite measure of objective social class for each household including the three main indicators: income, education, and occupational prestige [1,69].

*Income*. The annual household after-tax income was generated in the SOEP and given in Euro [80]. For the year 2009, SOEP households reported a mean annual after-tax income of 36,036.25 € (*SD* = 27,552.10). As expected, large households had higher incomes than small or single households. To overcome this problem, we adjusted for household size [81,82]. The formula for the OECD equivalence weights sets each single adult to 1.0, each additional adult to 0.7, and each child to 0.5. Thus, the weight of a four-person household including two children is 2.7 (for this specific household, the mean annual after-tax income would therefore be divided by 2.7). Next, due to the right skewness of the income variable and for better comparability, we applied the categorical scheme used in previous research [1]. We converted the category limits into Euro and divided them by the mean OECD equivalence weight of all households (which was about 1.8). The obtained category limits were rounded and resulted in the following categories: (1) < 6,000 €, (2) 6,001 € - 10,000 €, (3) 10,001 € - 14,000 €, (4) 14,001 € - 20,000 €, (5) 20,001 € - 30,000 €, (6) 30,001 € - 40,000 €, (7) 40,001 € - 60,000 €, (8) > 60,001 €. Households in our sample reported a mean category of 4.08 (*SD* = 1.48). We then standardized this income measure across all households.

*Education*. Education was assessed at the individual level using multiple items and was made available in categories that were based on the International Standard Classification of Education (ISCED-1997). The ISCED-1997 was originally developed by UNESCO [83] to differentiate between different internationally comparable educational levels (for an application in OECD countries, see the [84]). The categories are: (0) in school, (1) school dropout, (2) general elementary education, (3) middle vocational education, (4) vocational and postsecondary nontertiary education, (5) higher vocational education, or (6) higher education (for details, see [85]). Education at the household level was determined as the educational level of the household head (*M* = 3.87, *SD* = 1.47). We standardized education across all households.

*Occupational prestige*. Occupational prestige was rated at the individual level using the Standard International Occupational Prestige Scale (SIOPS; [86,87]). Employed participants reported their occupation, which was then converted to the SIOPS score (ranging from 0 to 100). As was done for education, occupational prestige at the household level was determined as the occupational prestige of the household head if he/she provided the respective information (*M* = 45.43, *SD* = 13.53). The score was further standardized across all households.

*Computation of objective social class*. According to previous research [69], we computed a composite measure of the objective social class of the household by averaging the standardized measures of income, education, and occupational prestige. If there was no information on one or two of the standardized measures, we used the mean of the remaining measure(s). Finally, the composite measure was z-standardized across all households (with a final range of -2.76 to 3.27).

#### Donation behavior

Donating was measured individually with two items in the year 2010. First, respondents were asked whether they had donated any money in the year 2009 (“Now we want to ask you about donations. In our understanding, donating is giving money for social, religious, cultural, charitable, and philanthropic purposes without expecting any kind of direct reward. This can be large amounts or even small amounts of money that you put in a donation box. Even the offertory in a church is a kind of donation. Did you donate any money in 2009—not taking into account membership subscriptions?”). Those who affirmed the first question were asked how much money they donated in 2009 (“How much money did you donate in the last year altogether?”). If at least one household member was a donor and affirmed the first question, the household was considered a donor household. In the present study, 53.51% (5,010 out of 9,363) of the households gave money to charity. Yet, only 4,907 of them reported how much they had given. As a measure of the relative monetary amounts of the donations, we summed up the individual donations made at the household level and determined the ratio of this sum to the annual household after-tax income. On average, the donating households gave 0.78% (*SD* = 1.64) of their annual after-tax income to charity. The mean of all households was 0.41% (*SD* = 1.25).

#### Analytical procedure

Because we wanted to investigate the influence of social class on donation behavior driven by the data as much as possible, we conducted all of our analyses in three steps.

In a first step, comparable to other studies [36,44], we separated the sample into deciles of objective social class or used the available categories of subjective social class to determine the proportion of prosocial actors and/or the mean value of prosociality per decile/category. The use of descriptive statistics gave us a first rough impression of the data.

In a second step, we computed locally weighted smoothing curves for the raw data (see [88], for an overview). Because of the different formats of our dependent variables, we applied two different kinds of local regressions with different fit criteria. For dichotomous variables (yes/no), we applied local likelihood fitting (Locfit; [89]; smoothing parameter = 1.0), which uses a local log-likelihood criterion (see also [90]). The Locfit curves illustrate the local probability of engaging in prosocial actions by social class. For metric variables, we applied local least squares fitting (LOESS; [91]; smoothing parameter = 0.8, polynomial = 1), which uses a local least squares criterion. Hence, the LOESS curves illustrate the “amount” of prosocial behavior by social class. On the basis of the curves that we fit, we could make preliminary assumptions about the relation between social class and donation behavior.

In a last step, we tested for statistical significance by computing logistic, ordinary least squares, and tobit regression analyses. Because previous research on the effects of social class on prosociality has found evidence of a negative relation, a positive relation, and a u-shaped relation in each of our studies, we also tested for curvilinear relations. To allow for a better interpretation of the regression coefficients, we also plotted the predicted values from our analyses.

### Results

**Fig 1:**
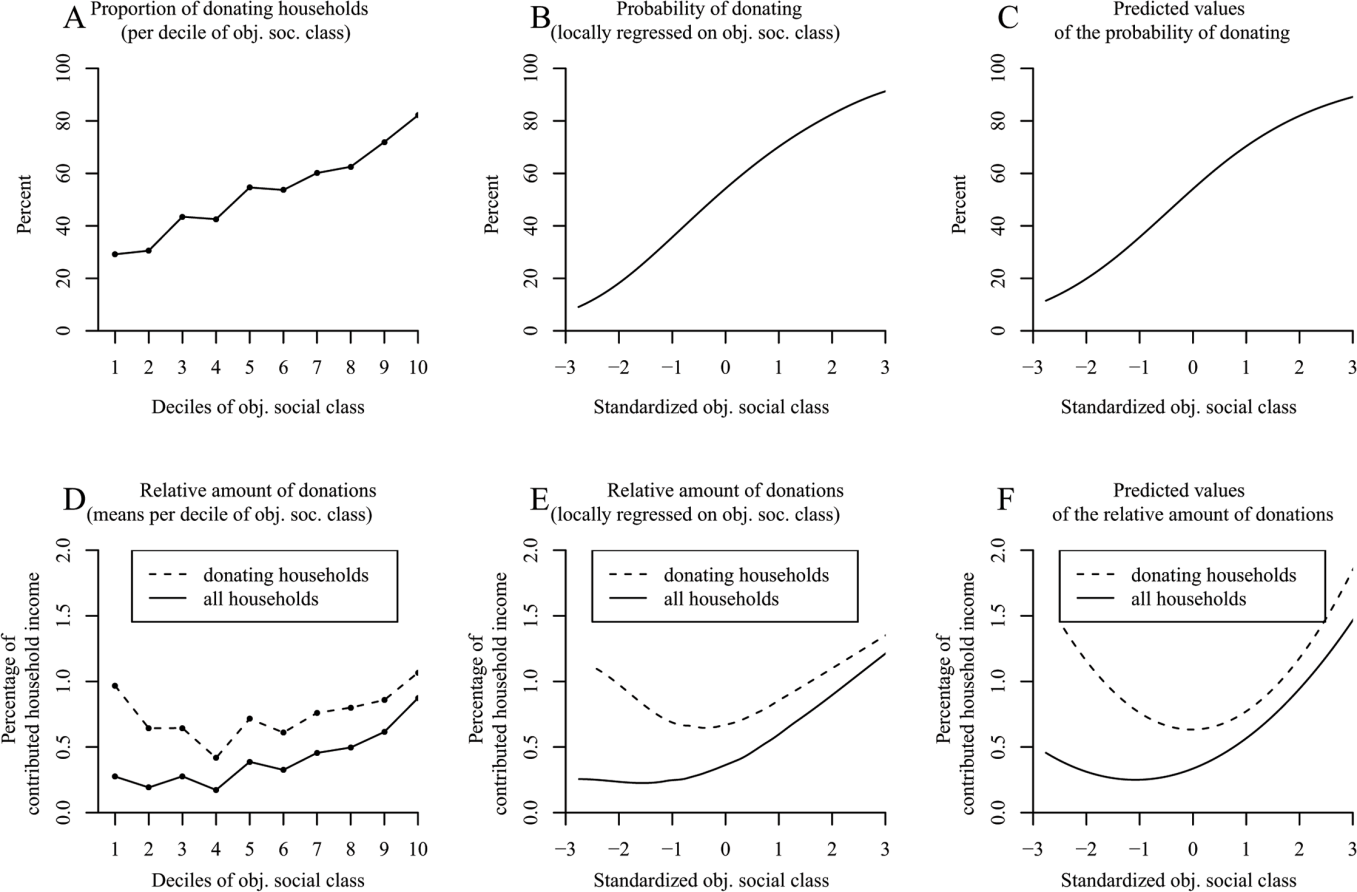
The positive effect of social class on donation behavior in the German SOEP (Study 1). Panel A shows the proportion of donating households per decile of social class. Panel B (*N* = 9,363 households) uses local likelihood fitting (Locfit) to adjust a curve to the raw data and illustrates the probability of donating by social class. Panel C shows the predicted values for the probability of donating determined via logistic regression. Panel D illustrates the amounts of the donations relative to household income per decile of social class. Panel E uses local least squares fitting (LOESS curves) for the relative amounts of the donations by social class. Panel F shows the predicted values for the relative amounts of donations determined via OLS regression. Panels D–F distinguish between a curve for donor households (*N* = 4,907 households) and a curve for all households (*N* = 9,260).

Comparable to other research [36], we found an increasing proportion of donating households with increasing deciles of social class (Fig 1A). We afterwards adjusted a Locfit curve to the raw data. Fig 1B also shows that increases in social class elevated the probability that a household would make charitable donations across the entire range of social class—albeit the slope seemed to be attenuated in the upper half of the distribution. In the following, we tested these observations for significance. The results of our logistic regression analysis as reported in Table 1 (column 1) revealed the distinct increase in the probability of donating in higher social classes—as can be seen in the high odds ratio for social class. Yet, results likewise confirmed the suspected attenuated slope—as can be seen in the odds ratio < 1 for squared social class (for a plot of the predicted values, see Fig 1C).

**Table 1:** Study 1: Effects of Social Class and its Quadratic Term on Donating (with Data from the German Socio-Economic Panel).

	Donation (yes/no)ª			Relative monetary amounts of donations for donor households only^b^			Relative monetary amounts of donations for all households^c^		
	*N*	*OR*	*z*	*N*	*b*	*t*	*N*	*b*	*t*
	9,363			4,907			9,260		
Objective social class		2.07	29.01***		.005	0.18		.158	11.47***
Objective social class^2^		0.97	-1.21		.133	6.08***		.073	6.39***

Objective social class was standardized across all households. *OR* = odds ratio; *b* = unstandardized regression coefficient.

^*a*^ Logistic Model (0 = nondonor; 1 = donor).

^b^ Nonlinear ordinary regression model computed excluding nondonors.

^c^ Nonlinear regression model including donor and nondonor households.

*** *p* < .001 (two-tailed).

Next, we analyzed the monetary amounts of the donations relative to the annual household income per decile of social class (dashed line in Fig 1D). Fig 1D suggests a u-shaped relation between donor households’ social class and the relative monetary amounts of donations with the highest decile donating the highest proportion of household income. In a second step, we fit a LOESS curve to the data (dashed line in Fig 1E), which also revealed a u-shaped relation between social class and the relative monetary amounts of the donations. The applied nonlinear ordinary least squares regression model confirmed the quadratic relation between donor households’ social class and the proportion of income donated (Table 1, column 2; see Fig 1F for a plot of the predicted values).

Because the main explanation put forth to account for the u curve had previously been that the analyses were restricted to donor households [43], we additionally conducted an examination of all households. When including both donor and nondonor households, we found an elevated curve for the relative monetary amounts per decile of social class (solid line in Fig 1D). This tendency also remained after fitting a LOESS curve to the raw data (solid line in Fig 1E). The corresponding ordinary least squares regression also revealed this nonlinear positive effect of social class on the relative monetary amounts of the donations (Table 1, column 3; see Fig 1F for a plot of the predicted values).

There is, however, another analysis that might be well-suited for the given data: a censored regression (also called a tobit regression; [92]). This analysis is most appropriate for data that are naturally censored (at zero). This procedure applies a maximum likelihood estimation and represents a mixture of the analysis on the probability of donating and the analysis on the relative monetary amounts of donations. When conducting the tobit regression for all households, we found a strong positive effect of social class (objective social class: *b* = .551, *t* = 22.16, *p* < .001; objective social class^2^: *b* = .011, *t* = 0.56, *p* = .58).

### Discussion

Taken together, the results of Study 1 clearly reveal that higher class households are more prosocial than lower social class households. Not only did a higher percentage of upper class households give anything to charity, but (if all households were included) they also gave a larger percentage of their income than the lower social class households.

However, the found effects on charitable donations might be specific for Germany due to the distinctive German social welfare system. By this system, those in need receive various social welfare benefits [63]. These are either tax-funded (e.g., the unemployment benefit II) or financed by the premiums of their members (e.g., the statutory nursing care insurance or the statutory health insurance). Because most citizens are compulsorily insured, the taxes and premiums are automatically collected from their monthly salaries by the state. The same applies to church taxes for the two institutional churches, the Catholic Church and the Evangelical Church, who use the money for their church welfare work. Thus, Germans may be accustomed to financing social welfare via taxes and charges and might see no further need to donate money to charity.

## Study 2: Effect of Social Class on Donating (CEX)

The objective of Study 2 was to check for a moderating role of the observed country. In Study 1, we revealed a positive effect of social class on donating in Germany. However, previous research on the relation between social class and prosociality was mostly conducted in countries with less elaborated social welfare systems such as the United States and Canada. It might be the case that social class has different effects on donation behavior in different countries. Therefore, in Study 2, we used data from the American CEX and conducted the same analyses as in Study 1.

### Method

#### Participants

CEX data were provided by the United States Department of Labor. On quarterly interview surveys, a reference person provides information on the household’s income and expenditures (including donations) from the last 3 months. Households are followed for a whole year. CEX data were available for the years 2005 to 2012, producing a total sample size of 79,907 households. Of these, 42,609 households were excluded because they missed at least one of the four interviews, 5,076 households were excluded because they gave incomplete income information, and 9 households were excluded because they made implausibly high donations (> 100% of their annual after-tax income). Comparable to previous research [36], we dropped another 123 households that provided a negative annual after-tax income. The remaining 32,090 households ranged in size from 1 to 14 persons (*M* = 2.55, *SD* = 1.50).

#### Objective social class

We intended to compute a composite measure of objective social class just as in Study 1. Yet, in the CEX, there is no information on a household’s occupational prestige, and there is information on the highest education of only the reference person. Thus, we created a composite measure by averaging (and afterwards z-standardizing) the standardized measures of the income and highest education of the reference person (or by taking only income if education was missing). The measure ranged from -2.41 to 2.21.

*Income*. In each quarterly interview, households provided information on the household’s income after taxes in the past 12 months. Therefore, we used the income information from only the last interview. In our sample, households reported a mean annual after-tax income of $65,492.31 (*SD* = 60,085.38). As in Study 1, we further adjusted for household size [81]. Because of the right skewness of the data and for better comparability, we further applied a previously applied categorical scheme [1] that was further adjusted for the mean OECD equivalence weight of all households (which was about 2.0). This resulted in the following categories: (1) < $7,500, (2) $7,501–$12,500, (3) $12,501–$17,500, (4) $17,501–$25,000, (5) $25,001–$37,500, (6) $37,501–$50,000, (7) $50,001–$75,001, (8) > $75,001. In our sample, CEX households reported a mean category of 4.55 (*SD* = 2.00). We standardized this income measure across our sample.

*Education*. The reference person’s education was assessed at the individual level in each interview using nine categories: (1) never attended school, (2) first through eighth grade, (3) ninth through 12th grade (no high school diploma), (4) high school graduate, (5) some college, less than college graduate, (6) associate’s degree (occupational/vocational or academic), (7) bachelor’s degree, (8) master’s degree, or (9) professional/doctoral degree. If reference persons gave inconsistent information on their highest education across the four interviews, education was set to missing. Reference persons reported a mean educational category of 5.27 (*SD* = 1.79). Education was standardized across the sample.

#### Donation behavior

In each of the four interviews, households reported a dollar amount for the contributions they made to charities. The total monetary amount of the donations was determined by summing up all household donations across the four interviews. If this sum was zero, the household was classified as a nondonor household. In our CEX sample, 43.89% (14,085 out of 32,090) of the households were donor households. We then calculated the ratio of this sum to the annual household after-tax income. On average, the donor households gave 0.89% (*SD* = 3.23) of their annual after-tax income to charity. Including all households, the mean relative amount of the donations was 0.39% (*SD* = 2.18).

In other studies using the CEX [36], sometimes contributions to political, educational, and religious organizations were considered as well. However, we decided to limit our analyses to contributions made to charities. This was done for the following reasons: On the one hand, contributions to political and educational organizations do not rule out some kind of (in)direct return. Hence, they may not have been completely prosocial. On the other hand, compared with Germany (and compared with our Study 1), contributions to religious organizations are spent differently in the US. That is, in the US, contributions are used to finance the religious community (e.g., for the pastor’s salary) and to fund charitable church activities. In Germany, however, the religious community is financed via church taxes and contributions, and the offertory is therefore almost exclusively put toward the church’s welfare work. Nevertheless, results for all kinds of donations can be found in the Supporting Information (S1 and S2 Tables).

### Results and Discussion

**Fig 2:**
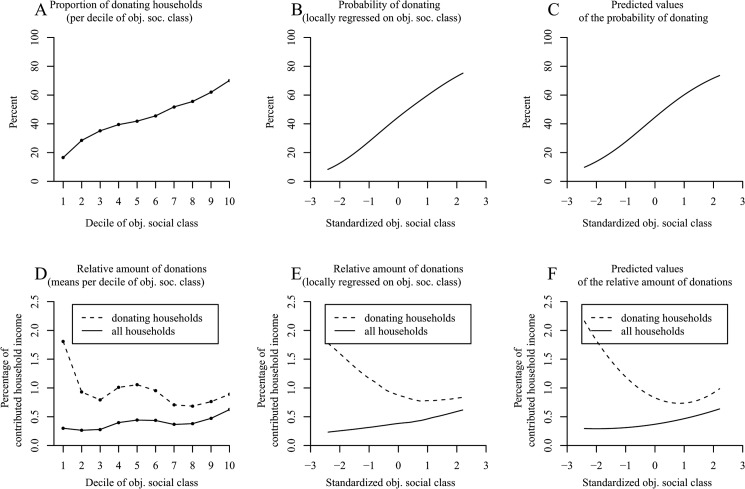
The positive effect of social class on donation behavior in the American Consumer Expenditure Survey (Study 2). Panel A shows the proportion of donating households per decile of objective social class. Panel B (*N* = 32,090 households) uses local likelihood fitting (Locfit) to adjust a curve to the raw data and illustrates the probability of donating by objective social class. Panel C shows the predicted values for the probability of donating determined via logistic regression. Panel D illustrates the amounts of the donations relative to household income per decile of objective social class. Panel E shows LOESS curves for the relative amounts of the donations by objective social class. Panel F shows the predicted values for the relative amounts of donations determined via OLS regression. Panels D–F distinguish between a curve for donor households (*N* = 14,084 households) and a curve for all households (*N* = 32,090 households).

Fig 2A unveils an increasing tendency to donate money to charity with elevations in the decile of social class. This positive linear tendency was corroborated by the Locfit curve in Fig 2B, and its statistical significance was verified with a logistic regression (Table 2, column 1; see Fig 2C for a plot of the predicted values).

**Table 2:** Study 2: Effects of Objective Social Class and its Quadratic Term on Donating (with Data from the American Consumer Expenditure Survey).

	Donation (yes/no)ª			Relative monetary amounts of donations for donor households only^b^			Relative monetary amounts of donations for all households^c^		
	*N*	*OR*	*z*	*N*	*b*	*t*	*N*	*b*	*t*
	32,090			14,085			32,090		
Objective social class		1.99	53.50***		-.228	-6.95***		.078	6.37***
Objective social class^2^		0.94	-4.94***		.135	4.98***		.020	1.79

Objective social class was standardized across all households. *OR* = odds ratio; *b* = unstandardized regression coefficient.

^*a*^ Logistic Model (0 = nondonor; 1 = donor).

^b^ Nonlinear ordinary regression model computed excluding nondonors.

^c^ Nonlinear regression model including donor and nondonor households.

*** *p* < .001 (two-tailed).

In terms of the relative monetary amounts of donations per decile of social class, Fig 2D (dashed line) shows a decreasing tendency that turns into an increase among the last three deciles. This exact trend—a decreasing tendency that turns into a slight increase—recurred in the applied LOESS curve (dashed line in Fig 2E) and was shown to be significant in a nonlinear ordinary least squares regression analysis (Table 2, column 2; the plotted predicted values are illustrated in Fig 2F).

However, just as in Study 1, when we repeated the preceding analyses using the donor and nondonor households together, we were able to rule out the possibility that the decreasing tendency was merely an artifact of limiting our sample to donor households. Interestingly, we found a relatively steady increase of the relative monetary amounts with ascending decile of social class (solid line in Fig 2D) and a steady increasing LOESS curve (solid line in Fig 2E). The corresponding ordinary least squares regression corroborated the positive linear effect of social class (Table 2, column 3; see Fig 2F for a plot of the predicted values).

Last, and as in Study 1, we conducted a tobit regression, an analysis that is specifically suited for data that are naturally censored at zero, to conjunctively examine the effects of social class on the probability of donating anything at all and on the relative monetary amounts of the donations. This overall analysis on donation behavior indicated a dominant positive effect of social class that mildly attenuated with elevated social class (objective social class: *b* = .898, *t* = 35.55, *p* < .001; objective social class^2^: *b* = -.118, *t* = -5.41, *p* < .001).

In sum, Study 2 confirmed the findings from Study 1 such that upper class households were more likely to donate anything to charity. When eliminating the artificial negative influence of social class on the relative amounts of the donations by investigating donor and nondonor households together, upper class households gave proportionately more than lower social class households. Moreover, in a combined analysis of donation behavior (tobit regression), social class showed a distinct positive effect on donating. Thus, all in all, the results were similar for the US and Germany such that higher class households were more prosocial than lower social class households.

## Study 3: Effect of Social Class on Donating (GSS)

The purpose of Study 3 was to replicate the effects found in Studies 1 and 2 and to further check for a moderating role of the measurement of social class. In our previous studies, we used only a measure of objective social class. As other researchers have pointed out, social class may also comprise an individual’s subjective standing in society [72,73]. In Study 3, we therefore used data from the American GSS with information on both objective and subjective social class. By doing so, we were able to investigate whether the effects of social class would diverge according to how social class was measured.

### Method

#### Participants

The GSS is an annual cross-sectional panel that interviews different persons in each year. In 2002, 2004, and 2012, respondents answered a question on their donation behavior. Educational, occupational, and income data as well as subjective social class data were obtained in the same years (only in 2012 was there no occupational data in terms of the ISCO-88). In total, 4,020 persons were asked about their donation behavior. Thirty persons did not answer the donation item, 15 persons did not provide the required demographic information, and a further 18 persons did not respond to the item asking about their subjective social class. Thus, the final sample included 3,975 persons (1,845 men; mean age = 46.87 years; *SD* = 17.37) for testing the effect of objective social class and a subsample of 3,957 persons for testing the effect of subjective social class.

#### Objective social class

We generated a composite measure of objective social class as we had in Study 1 using the three main indicators: income, education, and job prestige [69]. All indicators were asked for or generated annually in the GSS. The scores were z-standardized per year across the entire GSS sample (and ranged from -2.49 to 2.69).

*Income*. In 2002 and 2004, participants reported their total annual family income from all sources before taxes by choosing from 23 categories: (1) under $1,000 to (23) $110,000 or over. In 2012, the annual before-tax family income was assessed with 25 categories: (1) under $1,000 to (25) $150,000 or over. To maintain consistency, we intended to use the annual after-tax income that would have been weighted by family size. Because the GSS provided information only on before-tax income, we were forced to use this existing measure. Moreover, the assessment of before-tax income in categories prevented us from weighting income by family size.

Participants reported a mean income category of 16.72 (*SD* = 5.98) in 2012 and of 16.14 (*SD* = 5.42) in the other years. Income categories were standardized per year across the entire GSS sample.

*Education*. Respondents’ education was assessed with five categories: (0) less than high school, (1) high school, (2) associate/junior college, (3) bachelor’s degree, or (4) graduate degree. The mean educational category of our sample was 1.56 (*SD* = 1.20). Education was standardized per year across the entire GSS sample.

*Occupational prestige*. Respondents’ occupation was made available by the GSS according to the International Standard Classification of Occupations (ISCO-88; [93]). We transformed these categories into a SIOPS score ([86]; for further detail, see the Method section from Study 1). In our sample, respondents reported a mean SIOPS score of 43.46 (*SD* = 14.26). The score was further standardized for each year across the entire GSS sample.

#### Subjective social class

Respondents rated their subjective social class in each year according to four categories: (1) lower class, (2) working class, (3) middle class, or (4) upper class. Sample members reported a mean category of 2.43 (*SD* = 0.67). The score was further standardized for each year across the entire GSS sample. Subjective social class was significantly correlated with objective social class, *r* = .42, *p* < .001.

#### Donation behavior

Respondents were required to indicate how often they had given money to charity during the last 12 months. Answers were provided by choosing from six categories: (0) not at all in the past year, (1) once in the past year, (2) at least two or three times in the past year, (3) once a month, (4) once a week, or (5) more than once a week. In the GSS sample, 76.55% (3,043 out of 3,975) of the participants were donors (14.82% once in the past year, 33.21% at least two or three times in the past year, 18.11% once a month, 7.67% once a week, or 2.74% more than once a week).

#### Analytical procedure

The GSS data required some minor adjustments before we could use them in our analyses. First, because the GSS is an individual survey, we had to change our level of analysis from the household level to the individual level. Therefore, we also integrated the demographic covariates age (1 = male; 2 = female) and gender into our analysis because they have been shown to affect the probability of donating [8,38]. In addition, we entered the survey year into our regression models. Second, in the GSS, persons provided information only on whether or not they had given money to charity and, if they had, how often they had given money to charity. Similar to the previous studies, we therefore investigated the effects of social class on donation behavior in two separate approaches. We began by examining whether the probability of donating anything at all would be higher or lower with elevated social class. Then we investigated whether the frequency of donating was affected by social class by computing both ordered probit and ordinary least squares (OLS) regression models. Note that the frequency of donating is not equal to the actual amount of money donated and that this latter analysis is therefore not directly comparable to the two previous studies.

### Results and Discussion

**Fig 3:**
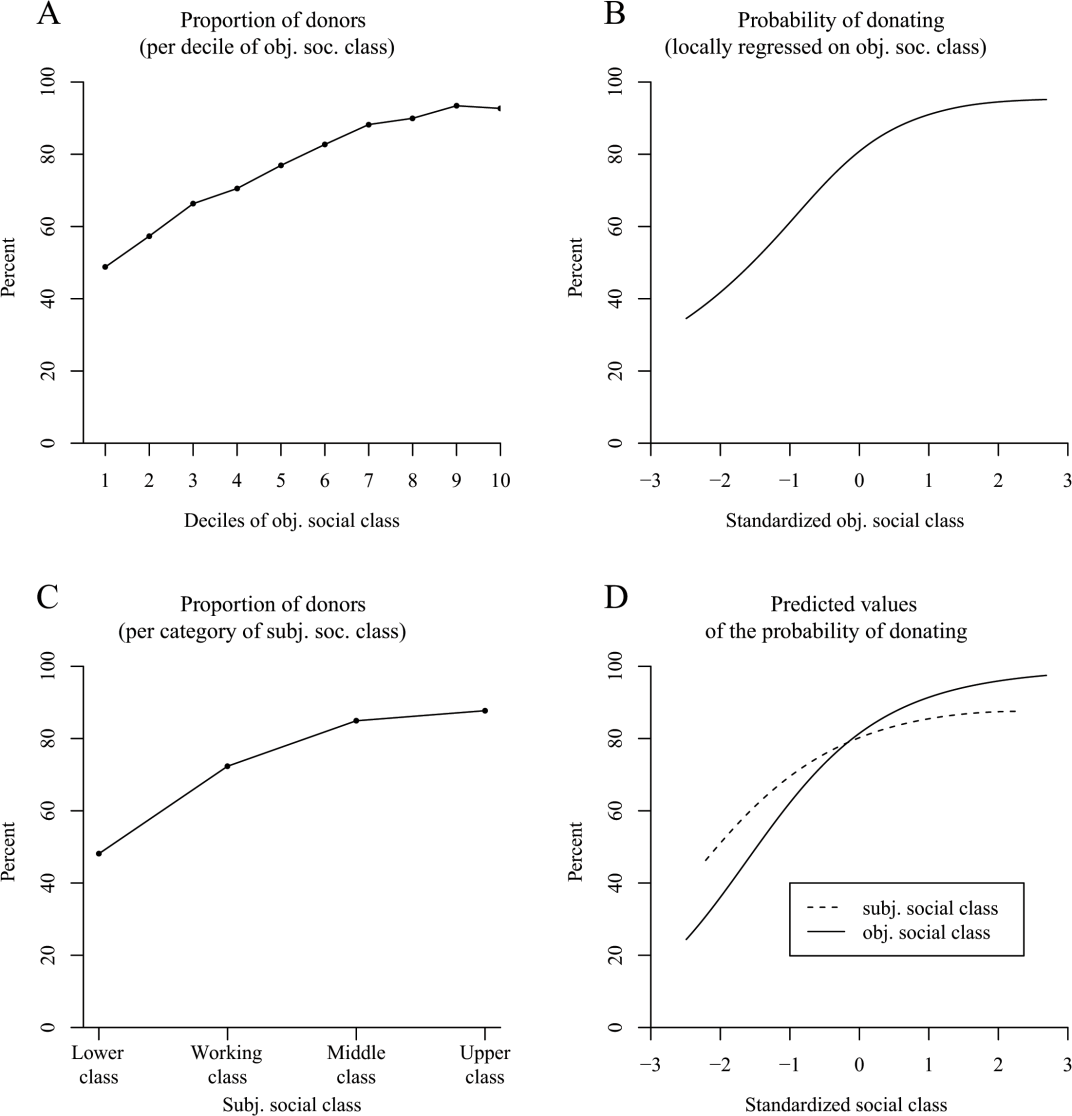
The positive effect of social class on the probability of donating in the American General Social Survey (Study 3). Panel A shows the proportion of donors per decile of objective social class. Panel B uses local likelihood fitting (Locfit) to adjust a curve to the raw data and illustrates the probability of donating by standardized objective social class (*N* = 3,975 persons). Panel C (*N* = 3,957 persons) shows the proportion of donors per category of subjective social class. Panel D illustrates the predicted values for the probability of donating determined via logistic regression. It distinguishes between a curve for subjective social class and a curve for objective social class.

The proportion of donors increased with elevated deciles of objective social class (Fig 3A) and with higher categories of subjective social class (Fig 3C). Because subjective social class was rated with only four categories, we omitted the fitting of locally weighted smoothing curves for this variable. However, in the objective social class sample, we fit the data with a Locfit curve that also revealed a higher probability of making charitable donations with increasing objective social class (Fig 3B). Yet, the slope weakened and seemed to be close to null among the higher social class individuals (which was most likely caused by a ceiling effect).

The logistic regression (which was based on the logits and, thus, could avoid the ceiling effect) that we conducted revealed a straight positive effect of objective social class on the probability of donating to charity (Table 3, Model 1, column 1; see the solid line in Fig 3D for the plotted predicted values). In addition, when we conducted the regression without the two covariates age and sex, the positive linear effect remained robust (objective social class: *OR* = 2.47, *z* = 18.11, *p* < .001; objective social class^2^: *OR* = 0.98, *z* = -0.59, *p* = .56). For subjective social class, the analyses revealed a positive linear effect that decreased with increasing social class (see the dashed line in Fig 3D for the plotted predicted values), an effect that was observed when the covariates were either integrated (Table 3, Model 2, column 1) or omitted (subjective social class: *OR* = 1.67, *z* = 12.08, *p* < .001; subjective social class^2^: *OR* = 0.93, *z* = -2.52, *p* < .05). In line with previous research, women and older persons were more likely to donate money to charity [8,38]. Aside from that, the probability of donating was smaller in 2012 compared with 2002 and 2004.

**Table 3:** Study 3: Separate Regressions for Donating on Objective Social Class (Model 1, N = 3,975) and Subjective Social Class (Model 2, N = 3,957) with Data from the American General Social Survey.

	Donating (yes/no)ª		Frequency of donating^b^ (Ordered probit model)		Frequency of donating^b^ (OLS regression model)	
	*OR*	*z*	*b*	*z*	*b*	*t*
**Model 1**						
Objective social class	2.54	18.29***	.392	22.31***	.447	23.04***
Objective social class^2^	0.95	-1.15	-.064	-4.50***	-.058	-3.58***
Gender	1.33	3.50***	.061	1.81	.068	1.74
Age	1.02	10.35***	.014	14.17***	.016	14.36***
Year						
2002 (*N* = 1,354)						
2004 (*N* = 1,333)	1.07	0.63	.055	1.35	.064	1.35
2012 (*N* = 1,296)	0.65	-4.41***	-.174	-4.21***	-.199	-4.19***
**Model 2**						
Subjective social class	1.61	11.16***	.230	13.08***	.274	13.11***
Subjective social class^2^	0.90	-3.36**	-.039	-2.89**	-.038	-2.40*
Gender	1.25	2.80**	.034	1.01	.038	0.94
Age	1.02	9.01***	.012	11.79***	.014	12.01***
Year						
2002 (*N* = 1,349)						
2004 (*N* = 1,328)	1.07	0.73	.052	1.27	.065	1.31
2012 (*N* = 1,287)	0.66	-4.32***	-.173	-4.19***	-.205	-4.12***

Objective social class and subjective social class were standardized across all subjects separately for each year. Reference value for year is 2002. *OR* = odds ratio. *b* = estimated coefficient of the ordered probit model. Gender: 1 = male; 2 = female.

^*a*^ Logistic regresison (0 = nondonor; 1 = donor).

^b^ 0 = not at all in the past year; 5 = more than once a week.

* *p* < .05.

** *p* < .01.

*** *p* < .001 (two-tailed).

**Fig 4:**
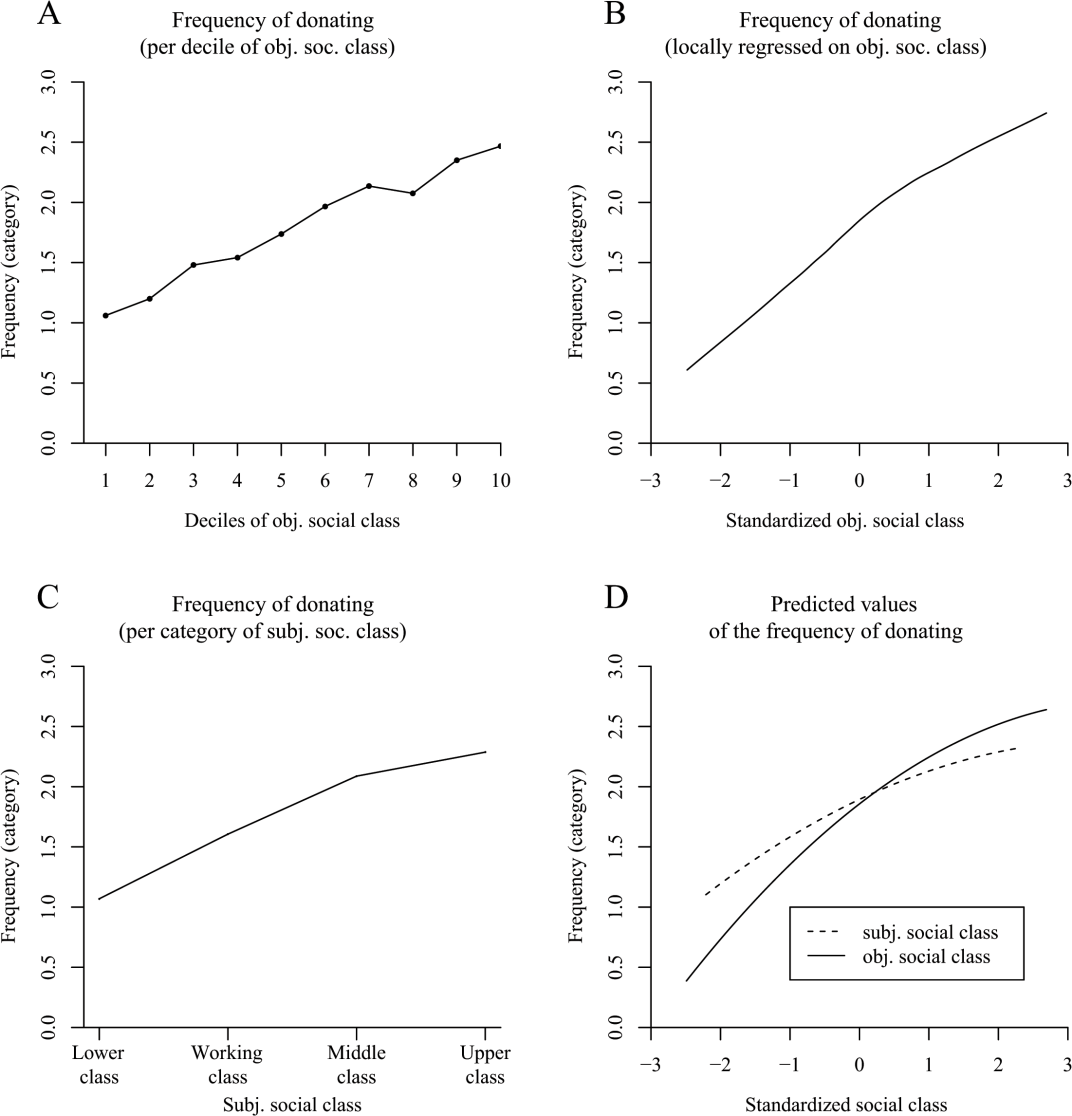
The positive effect of social class on the frequency of donating in the American General Social Survey (Study 3). Panels A–D illustrate the frequency of donating based on six categories (0 = not at all in the past year, 5 = more than once a week). Panel A shows the frequency of donating per decile of objective social class. Panel B uses local least squares fitting (LOESS curve) to adjust a curve to the raw data and illustrates the frequency of donating by standardized objective social class (*N* = 3,975 persons). Panel C (*N* = 3,957 persons) shows the frequency of donating per category of subjective social class. Panel D illustrates the predicted values for the frequency of donating determined via OLS regression. It distinguishes between a curve for subjective social class and a curve for objective social class.

In terms of the frequency of donating, Fig 4A and 4B indicate a positive effect of objective social class. This observation was confirmed by ordered probit and OLS regression models that showed a positive nonlinear effect of social class (Table 3, Model 1, columns 2 and 3; see the solid line in Fig 4D for the plotted predicted values from the OLS regression). The same effect was also observed in an analysis without the covariates age and sex (ordered probit model: objective social class: *b* = .379, *z* = 21.76, *p* < .001; objective social class^2^: *b* = -.054, *z* = -3.81, *p* < .001; OLS regression model: objective social class: *b* = .446, *t* = 22.44, *p* < .001; objective social class^2^: *b* = -.048, *t* = -2.93, *p* < .01). According to Fig 4C, an elevated subjective social class also seemed to increase the frequency of donating. Again, a positive nonlinear effect was found to be significant both with the covariates age and sex (Table 3, Model 2, columns 2 and 3; see the dashed line in Fig 4D for the plotted predicted values from the OLS regression) and without the covariates in the ordered probit model (subjective social class: *b* = .254, *z* = 14.64, *p* < .001; subjective social class^2^: *b* = -.033, *z* = -2.44, *p* < .05; OLS regression model: subjective social class: *b* = .309, *t* = 14.70, *p* < .001; subjective social class^2^: *b* = -.030, *t* = -1.87, *p* = .06).

Taken together, Study 3 replicated the results of the previous studies: Higher social class individuals were more likely to make charitable donations and they made them more frequently than people in the lower social realms. This effect was independent of whether social class was considered to be a combination of objective indicators of socioeconomic status (objective social class) or a subjective ranking with respect to other people (subjective social class).

## Study 4: Effect of Social Class on Volunteering (SOEP)

In the first three studies, we assessed prosocial behavior only in the form of the transfer of money. The goal of Study 4 was to extend the level of analysis to another important form of charity: volunteering. This is important to do as one might argue that people in the lower social classes who suffer from money scarcity are less likely to give money to charity. Yet, because a higher proportion of lower social class individuals are unemployed or employed only part-time [94], they have time resources and, thus, more time to volunteer. Some studies have found that part-time workers and unemployed individuals more often volunteer [11,41,95]. On the other hand, less educated [11] and lower income people [10,40] have shown lower rates of volunteer work. In Study 4, we wanted to shed further light on this issue.

### Method

#### Participants

Study 4 used data from the German SOEP (Version 29). Data on volunteering were gathered in the years 2005, 2007, 2009, and 2011. Educational and occupational data were obtained in the same years and income data in the following years. Because the SOEP is a longitudinal panel, our sample consisted of 33,072 persons (15,817 men) who were asked about their volunteering once to four times (mean = 2.51 times; making a total of 82,966 observations). On average, the respondents were 49.43 years (*SD* = 17.63).

#### Volunteering

In the SOEP, respondents had to use a single item to rate how often they volunteered for associations, organizations, or social services in their leisure time. Ratings were made by choosing from four categories: (0) never, (1) less frequently, (2) every month, or (3) every week. Across our observations, 31.75% of the sample were volunteers (9.97% every week, 8.76% every month, 13.02% less frequently).

#### Objective social class

We assessed objective social class as we did in Study 1 but on the individual level. Participants reported a mean educational category (ISCED-1997) of 3.68 (*SD* = 1.43), a mean occupational prestige score (SIOPS) of 44.28 (*SD* = 13.38), and a mean household income category of 4.00 (*SD* = 1.46; we preferred household income to individual income because otherwise spouses of high-income participants would be allocated to a lower social class than their spouses). The final score ranged from -2.39 to 3.50.

#### Analytical procedure

Similar to how we analyzed donation behavior, we first examined the effects of social class on the probability of volunteering and, second, on the frequency of volunteering. Because the SOEP is a longitudinal panel and some of the respondents gave information on their social class and volunteering in more than one year, we applied multilevel logistic, ordered probit, and ordinary regression models with repeated measurements nested within persons.

In addition, because gender and age were shown to substantially influence the probability of volunteering [11,96,97], we controlled for age and gender (1 = male; 2 = female) in our analysis.

### Results and Discussion

**Fig 5:**
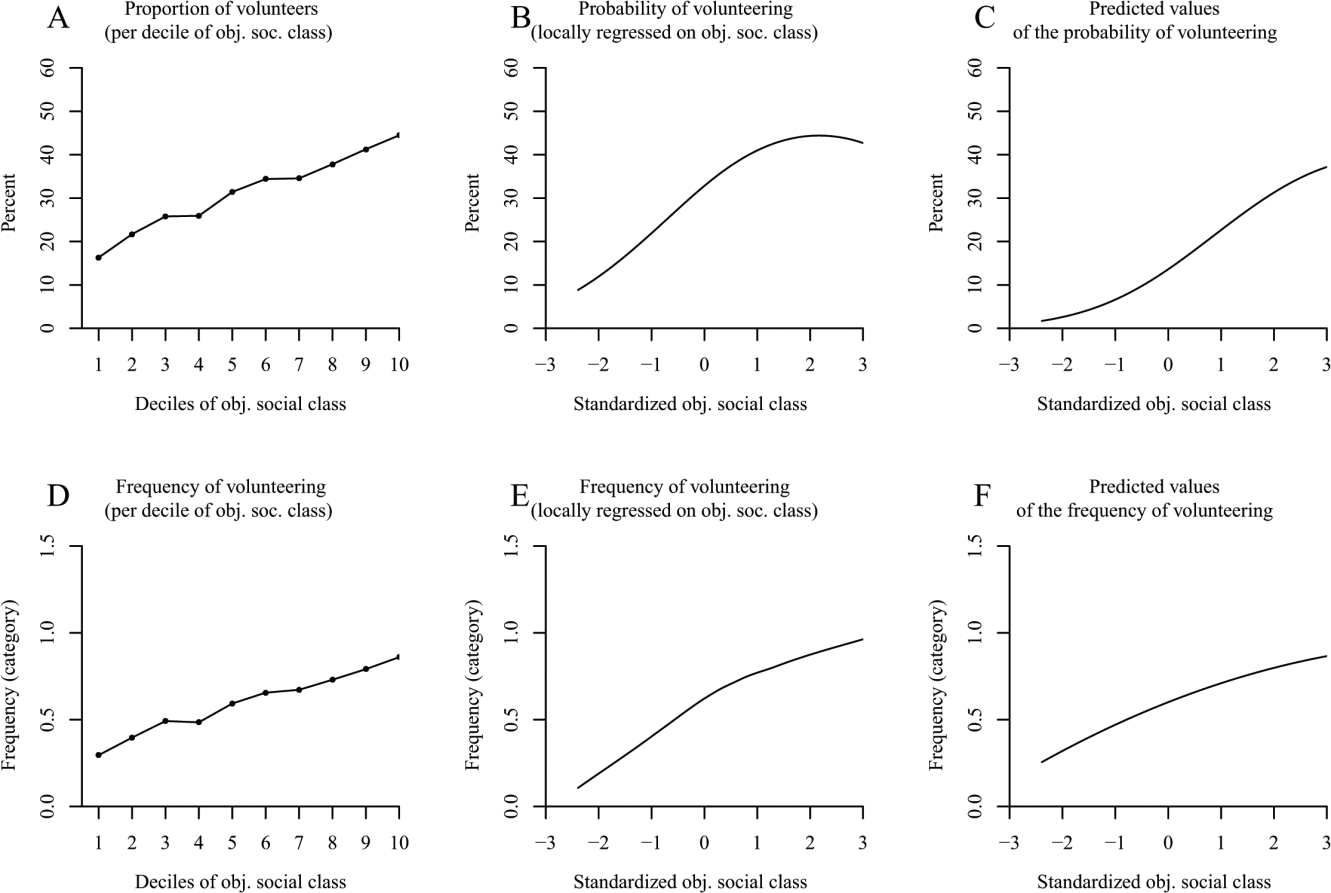
The positive effect of social class on volunteering in the German SOEP (Study 4; based on N = 33,072 persons and 82,966 observations). Panel A shows the proportion of volunteers per decile of objective social class. Panel B uses local likelihood fitting (Locfit) to adjust a curve to the raw data and illustrates the probability of volunteering by objective social class. Panel C shows the predicted values for the probability of volunteering determined via logistic regression. Panels D–F illustrate the frequency of volunteering based on four categories (0 = never, 3 = every week). Panel D shows the mean frequency of volunteering per decile of objective social class. Panel E shows a LOESS curve for the frequency of volunteering by objective social class. Panel F illustrates the predicted values for the frequency of volunteering determined via multilevel ordinary regression.

A determination of the proportion of volunteers per decile of social class pointed toward a positive relation between social class and volunteering (Fig 5A). This first impression was sustained when we fit Locfit curves to the raw data. The curve in Fig 5B indicates that the probability of volunteering increased with elevated social class—but the slope appeared to reflect a mild decline at the higher levels of social class.

As can be seen in Table 4 (column 1), the multilevel logistic regression confirmed the increased probability of volunteering with elevated social class (observable in the substantially high odds ratios for social class). In addition, we found a quadratic effect indicating that the slope indeed changed significantly to reflect a decline at the higher levels of social class (observable in the odds ratios of squared social class < 1; see Fig 5C for a plot of the predicted values). When conducting the same analysis without the covariates age and sex, the effect remained robust (objective social class: *OR* = 2.03, *z* = 32.51, *p* < .001; objective social class^2^: *OR* = 0.92, *z* = -5.57, *p* < .001). Contrary to previous research [11,97], men were more likely to volunteer than women, a finding that might be due to a large volunteer sector in sports such as soccer in Germany. We also found an effect of age on volunteering, pointing toward a decrease in volunteer work for older people. Moreover, there were significant differences in the mean rate of volunteering between years of measurement, even though volunteering was quite stable within persons across the different years of measurement, *ICC* = .763, χ²(1) = 17,718.2, *p* < .001.

**Table 4:** Study 4: Effects of Objective Social Class on Volunteering in the German SOEP.

	Volunteering (yes/no)ª		Frequency of volunteering^b^ (Multilevel ordered probit model)		Frequency of volunteering^b^ (Multilevel ordinary regression model)	
	*OR*	*z*	*b*	*z*	*b*	*z*
Objective social class	2.03	32.49***	.336	29.10***	.120	26.52***
Objective social class^2^	0.91	-5.70***	-.048	-5.88***	-.010	-3.25**
Gender	0.66	-9.54***	-.273	-11.08***	-.117	-11.79***
Age	0.98	-13.65***	-.008	-10.93***	-.002	-6.05***
Year						
2005 (*N* = 20,881)						
2007 (*N* = 20,640)	0.87	-4.34***	-.048	-3.06**	-.012	-1.88
2009 (*N* = 20,564)	0.81	-6.33***	-.074	-4.47***	-.021	-3.17**
2011 (*N* = 20,881)	1.14	3.81***	.067	3.97***	.027	3.93***

82,966 observations were nested within 33,072 persons. Objective social class was standardized per year across all subjects. The reference value for year was 2005. *OR* = Odds Ratio. *b* = estimated coefficient of the multilevel ordered probit model. Gender: 1 = male; 2 = female.

^*a*^ Multilevel logistic regression model (0 = nonvolunteer; 1 = volunteer).

^b^ 0 = never, 3 = every week.

** *p* < .01.

*** *p* < .001 (two-tailed).

In terms of the frequency of volunteering, Fig 5D indicates an increased frequency of volunteering with each elevated decile of social class. The LOESS curve in Fig 5E corroborates this finding for the standardized objective measure of social class. Last, we computed both a multilevel ordered probit regression and a multilevel ordinary regression for continuous dependent variables using the original answer categories (Table 4, columns 2 and 3). Same as for the likelihood of volunteering, elevated social class also positively affected the frequency of volunteering. However, this positive effect diminished with increasing social class (see Fig 5F for the plotted predicted values from the multilevel ordinary regression model). Moreover, it was independent of whether or not the covariates age and sex were entered into the model (Table 4, columns 2 and 3) or not (multilevel ordered probit model: objective social class: *b* = .337, *z* = 29.22, *p* < .001; objective social class^2^: *b* = -.047, *z* = -5.74, *p* < .001; multilevel ordinary regression model: objective social class: *b* = .122, *z* = 26.95, *p* < .001; objective social class^2^: *b* = -.010, *z* = -3.11, *p* < .01).

All in all, Study 4 demonstrated that the probability and frequency of volunteering increased with elevated social class. Thus, in our large and representative German sample, higher class persons were not only more willing to give money than lower social class individuals, but they were also more willing to give their time.

## Study 5: Effect of Social Class on Volunteering (GSS)

In Study 5, we attempted to replicate the positive effect of social class found in Study 4 in another country: the United States. In addition, by using data from the American GSS, we were able to check for a moderating role of the measure of social class (objective vs. subjective). As we had in Study 4, we investigated whether social class affected the probability of volunteering at all and whether or not there were effects of social class on the frequency of volunteering.

### Method

#### Participants

Respondents were asked about their volunteering, education, occupation, family income, and subjective social class in 2002, 2004, and 2012 (but there were no occupational data available for 2012). In sum, 4,020 persons were asked about their volunteering. Twenty-two persons did not provide the corresponding information, 15 persons did not provide the required demographic information, and a further 19 persons did not respond to the item asking about their subjective social class. Thus, the final sample included 3,983 persons (1,848 men; mean age = 46.89 years; *SD* = 17.38) for testing the effect of objective social class and a subsample of 3,964 persons for testing the effect of subjective social class.

#### Objective and subjective social class

Measures of objective and subjective social class were generated in the same way as in Study 3. Participants reported a mean before-tax family income category of 16.72 (*SD* = 5.98) in 2012 and of 16.14 (*SD* = 5.42) in the other years, a mean educational category of 1.56 (*SD* = 1.20), a mean occupational prestige score of 43.44 (*SD* = 14.27), and a mean subjective social class category of 2.43 (*SD* = 0.67). The final score for objective social class ranged from -2.49 to 2.69. The measures of subjective and objective social class were significantly correlated, *r* = .42, *p* < .001.

#### Volunteering

Sample members reported how often they had done volunteer work for a charity during the last 12 months. They answered according to six categories: (0) not at all in the past year, (1) once in the past year, (2) at least two or three times in the past year, (3) once a month, (4) once a week, or (5) more than once a week. In our sample, 46.42% (1,849 out of 3,983) of the participants were volunteers (12.10% once in the past year, 16.67% at least two or three times in the past year, 8.79% once a month, 4.39% once a week, and 4.47% more than once a week).

### Results and Discussion

**Fig 6:**
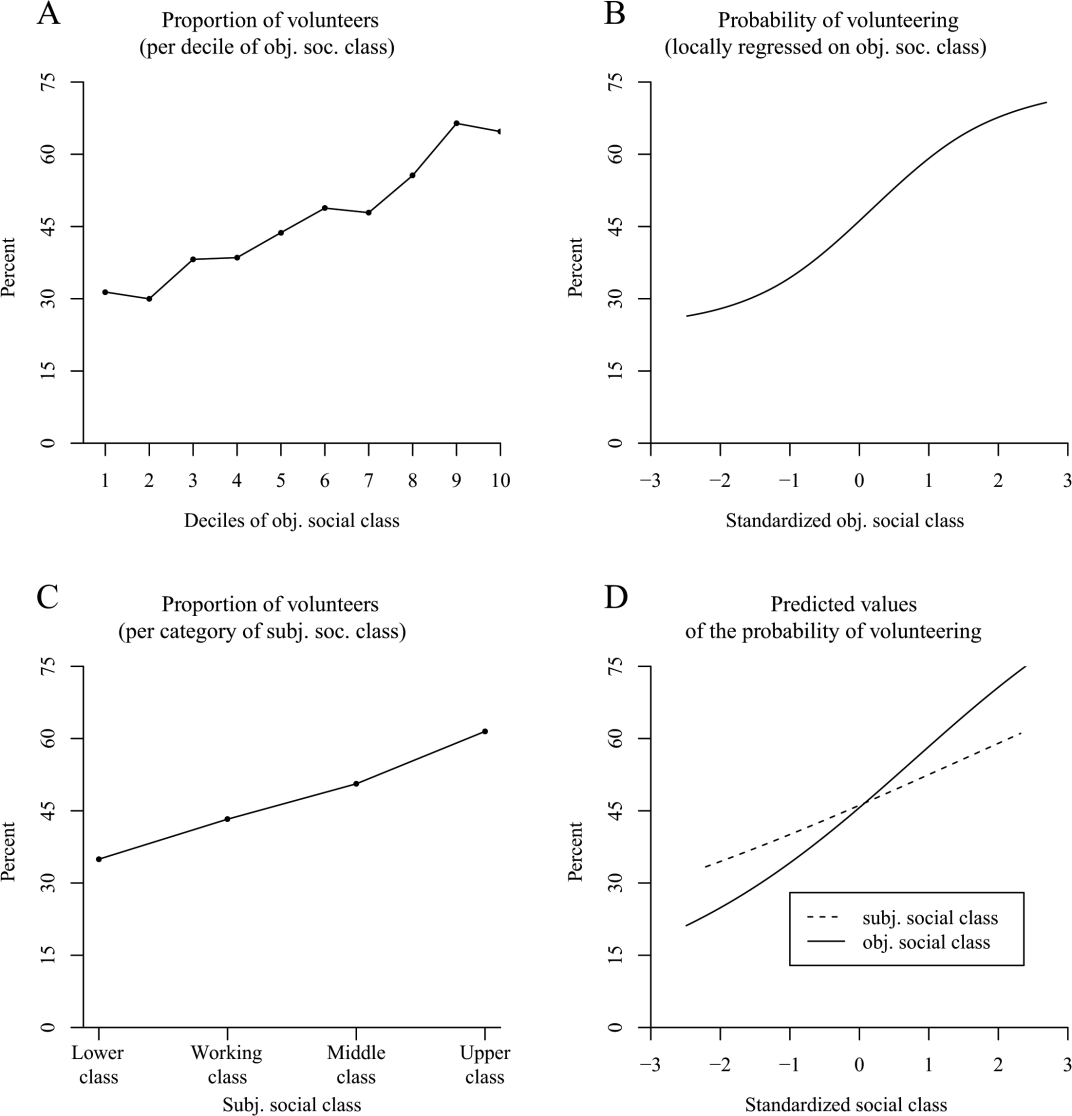
The positive effect of social class on the probability of volunteering in the American General Social Survey (Study 5). Panel A shows the proportion of volunteers per decile of objective social class. Panel B uses local likelihood fitting (Locfit) to adjust a curve to the raw data and illustrates the probability of volunteering by standardized objective social class (*N* = 3,983 persons). Panel C (*N* = 3,964 persons) shows the proportion of volunteers per category of subjective social class. Panel D illustrates the predicted values for the probability of volunteering determined via logistic regression. It distinguishes between a curve for subjective social class and a curve for objective social class.

Fig 6A shows that the proportion of volunteers increased with elevated deciles of objective social class. Applying a local regression to the raw data, we further found an increase in the probability of volunteering with increasing objective social class (Fig 6B). This linearity turned out to be significant when we conducted logistic regression analyses both with the covariates age and sex (Table 5, Model 1, column 1; see the solid line in Fig 6D for the plotted predicted values) and without the covariates (objective social class: *OR* = 1.62, *z* = 14.27, *p* < .001; objective social class^2^: *OR* = 1.01, *z* = 0.36, *p* = .72).

**Table 5:** Study 5: Separate Regressions for Volunteering on Objective Social Class (Model 1, N = 3,983) and Subjective Social Class (Model 2, N = 3,964) with Data from the American General Social Survey.

	Volunteering (yes/no)ª		Frequency of volunteering^b^ (Ordered probit model)		Frequency of volunteering^b^ (OLS regression model)	
	*OR*	*z*	*b*	*z*	*b*	*t*
**Model 1**						
Objective social class	1.64	14.50***	.248	13.49***	.291	12.86***
Objective social class^2^	1.02	0.53	.017	1.15	.047	2.50*
Gender	1.33	4.34***	.170	4.68***	.212	4.65***
Age	0.99	-4.19***	-.001	-0.71	.001	0.71
Year						
2002 (*N* = 1,354)						
2004 (*N* = 1,333)	1.22	2.51*	.111	2.52*	.132	2.40*
2012 (*N* = 1,296)	1.04	0.50	.041	0.92	.058	1.04
**Model 2**						
Subjective social class	1.29	7.46***	.135	7.25***	.164	6.85***
Subjective social class^2^	1.00	0.15	.008	0.55	.020	1.10
Gender	1.28	3.79***	.152	4.20***	.193	4.15***
Age	0.99	-4.98***	-.001	-1.38	.000	-0.05
Year						
2002 (*N* = 1,349)						
2004 (*N* = 1,328)	1.22	2.50*	.109	2.49*	.131	2.33*
2012 (*N* = 1,287)	1.04	0.49	.040	0.90	.056	0.98

Objective social class and subjective social class were standardized across all subjects separately for each year. The reference value for year was 2002. *OR* = odds ratio. *b* = estimated coefficient of the multilevel ordered probit model. Gender: 1 = male; 2 = female.

^*a*^ Logistic regression (0 = nonvolunteer; 1 = volunteer).

^b^ 0 = not at all in the past year; 6 = more than once a week.

* *p* < .05.

*** *p* < .001 (two-tailed).

**Fig 7:**
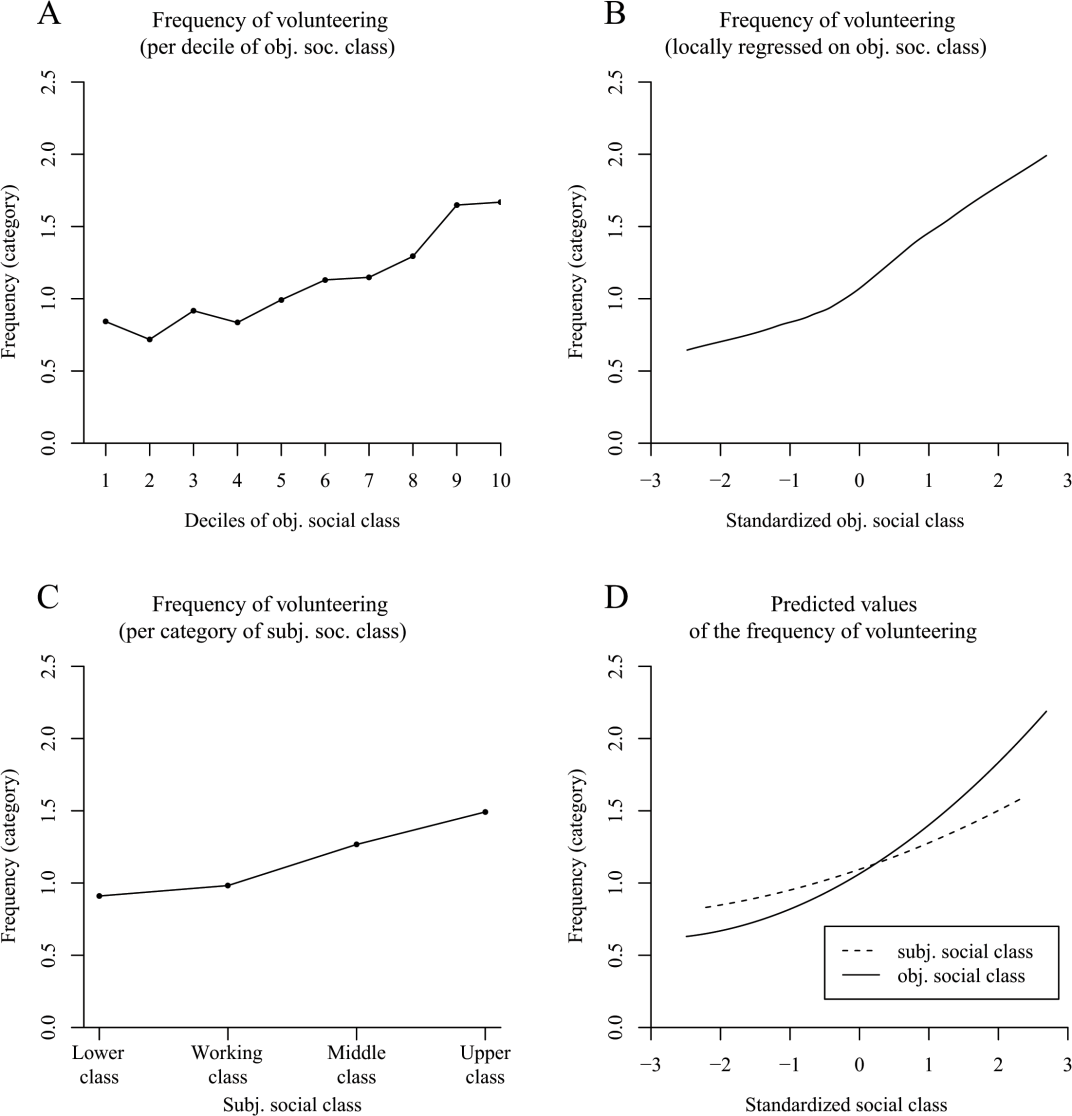
The positive effect of social class on the frequency of volunteering in the American General Social Survey (Study 5). Panels A–D illustrate the frequency of volunteering using six categories (0 = not at all in the past year, 5 = more than once a week). Panel A shows the frequency of volunteering per decile of objective social class. Panel B uses local least squares fitting (LOESS curve) to adjust a curve to the raw data and illustrates the frequency of volunteering by standardized objective social class (*N* = 3,983 persons). Panel C (*N* = 3,964 persons) shows the frequency of volunteering per category of subjective social class. Panel D illustrates the predicted values for the frequency of volunteering determined via OLS regression. It distinguishes between a curve for subjective social class and a curve for objective social class.

Moreover, the positive effect remained when we focused on the frequency rather than the probability of volunteering. The graphs illustrate a rising curve for the frequency of volunteering with elevated deciles of objective social class (Fig 7A) and in the locally weighted analysis (Fig 7B). This positive effect turned out to be significant when we computed ordered probit and OLS regressions both with the covariates age and sex (Table 5, Model 1, columns 2 and 3; see the solid line in Fig 7D for the plotted predicted values of the OLS regression) and without the covariates (ordered probit model: objective social class: *b* = .244, *z* = 13.27, *p* < .001; objective social class^2^: *b* = .016, *z* = 1.09, *p* = .28; OLS regression model: objective social class: *b* = .287, *t* = 12.63, *p* < .001; objective social class^2^: *b* = .048, *t* = 2.52, *p* < .05).

For the effect of subjective social class, we found an increase in the proportion of volunteers with elevated subjective social class (Fig 6C). This effect was significant in the logistic regression (Table 5, Model 2, column 1; see the dashed line in Fig 6D for the plotted predicted values) and also remained when the logistic regression was conducted without the covariates age and sex (subjective social class: *OR* = 1.25, *z* = 6.75, *p* < .001; subjective social class^2^: *OR* = 1.00, *z* = -0.05, *p* = .96).

In terms of the frequency of volunteering, the frequency seemed to increase with higher categories of subjective social class (Fig 7C). The ordered probit and OLS regressions confirmed the expected positive effect in analyses with the covariates age and sex (Table 5, Model 2, columns 2 and 3; see the dashed line in Fig 7D for the plotted predicted values) and without the covariates (ordered probit model: subjective social class: *b* = .129, *z* = 7.01, *p* < .001; subjective social class^2^: *b* = .007, *z* = 0.47, *p* = .64; OLS regression model: subjective social class: *b* = .162, *t* = 6.82, *p* < .001; subjective social class^2^: *b* = .020, *t* = 1.08, *p* = .28).

Comparable to previous research [11,97] but in contrast to the study in Germany (Study 4), women were more likely to volunteer and volunteered more often. Similar to Study 4, older people had a reduced probability of volunteering, and volunteering varied across the years. In sum, Study 5 replicated the positive effect of social class on volunteering in a representative US sample.

## Study 6: Effect of Social Class on Volunteering for Charitable Activities (ISSP)

In our studies, we found that people in the higher social realms were more likely to volunteer for a charity and that this effect occurred in Germany (Study 4) as well as in the United States (Study 5). To examine whether there would be differences in the effect of social class on volunteering in other countries, we expanded our research to more than two countries and to some nonwestern countries [66]. In Study 6, we analyzed data from the ISSP, an annual program of cross-national surveys comprising more than 30 countries. In 1998, the ISSP contained an item on volunteering as well as items on subjective social class and the indicators of objective social class. Thus, we were able to test whether country and the measure of social class (objective vs. subjective) were moderators of the relation between social class and volunteering. As we did in Studies 4 and 5, we analyzed the effects of social class on both the probability and the frequency of volunteering.

### Method

#### Participants

In 1998, the ISSP consisted of surveys in 31 countries. Program members were asked about their volunteering, subjective social class, income, education, and occupation. One country (Northern Ireland) was excluded from our study because respondents gave information on only one indicator of social class (education). In total, 37,307 persons reported on their volunteering. Of these, 82 persons did not respond to the questions about the indicators of their objective social class and an additional 91 persons did not give the required demographic information. The final sample for testing the effect of objective social class therefore included 37,136 persons (17,249 men; mean age = 45.14, *SD* = 17.12) who were nested within 30 countries. In two countries (Netherlands, Great Britain), respondents were not asked for their subjective social class, and a further 2,156 persons did not answer the corresponding question in the other countries. Thus, the subsample for testing the effect of subjective social class included 32,257 persons (15,073 men; mean age = 45.25, *SD* = 17.04) who were nested within 28 countries.

#### Objective social class

Identical to the other studies, a measure of objective social class was computed by averaging the standardized measures of income, education, and occupational prestige [69]. The average was calculated for any viable combination of the three indicators and z-standardized per country (ranging from -3.64 to 5.51).

*Income*. Participants reported their family income in their countries’ currency. There was, however, a large amount of heterogeneity in the income measure. Whereas respondents in some countries were asked for net income (e.g., Czech Republic, Germany), respondents in other countries were asked for their income before taxes (e.g., Denmark, Cyprus), or else the question did not specify the kind of income (e.g., Australia, Canada). In addition, some countries presented annual income (e.g., Japan, United States), whereas others presented monthly income (e.g., Italy, Poland). We were therefore not able to apply the categorical scheme used in previous research [1]. However, to account for the skewness of the income measure, we took the logarithm of each countries’ income measure. Afterwards, the logarithmized income was adjusted for household size [81] and standardized per country.

*Education*. Respondents indicated their highest education by choosing from one of seven educational categories: (0) none, still in school, (1) incomplete primary, (2) primary completed, (3) incomplete secondary, technical school, (4) secondary completed, (5) incomplete + complete semi-higher qualification, incomplete university, others, or (6) university completed. Persons who were still in school were set to missing. The mean educational category of our sample was 3.63 (*SD* = 1.38). Education was standardized per country.

*Occupational prestige*. Occupational prestige was made available by the ISSP according to the ISCO-88 [93]. These categories were further transformed into a SIOPS score [86]. In our sample, respondents reported a mean SIOPS score of 41.44 (*SD* = 13.22). The score was standardized per country.

#### Subjective social class

Respondents rated their subjective social class by choosing from six categories: (1) lower class, (2) working class, (3) lower middle class/upper working class, (4) middle class, (5) upper middle class, or (6) upper class. In Australia, Japan, and the United States, ratings were made with only four categories (1, 2, 4, and 6). Respondents reported a mean category of 3.18 (*SD* = 1.19). The score was standardized per country.

#### Volunteering

Respondents were asked about their volunteering (“During the last 12 months, did you do volunteer work in charitable activities [helping the sick, elderly, poor, etc.]? Voluntary activity is unpaid work, not just belonging to an organization or group. It should be of service or benefit to other people or the community and not only to one’s family or personal friends”). They answered this question by choosing from four categories: (0) no, (1) yes, once or twice, (2) yes, 3–5 times, or (3) yes, 6 or more times. Overall, 25.57% (9,496 out of 37,136) of our sample were volunteers (12.84% yes, once or twice, 4.94% yes, 3–5 times, 7.80% yes, 6 or more times).

#### Analytical procedure

Due to the specific features of the ISSP, there were two minor adjustments that we made to our analyses. First, because the respondents in our sample were nested within countries, we applied multilevel logistic, ordered probit, and ordinary regression models with Level 1 representing the respondent and Level 2 representing the respondent’s country. Second, to test for different effects of social class in divergent countries, we allowed the effects of social class and squared social class to vary between countries. Analogous to the previous studies, we also controlled for gender (1 = male; 2 = female) and age.

### Results and Discussion

**Fig 8:**
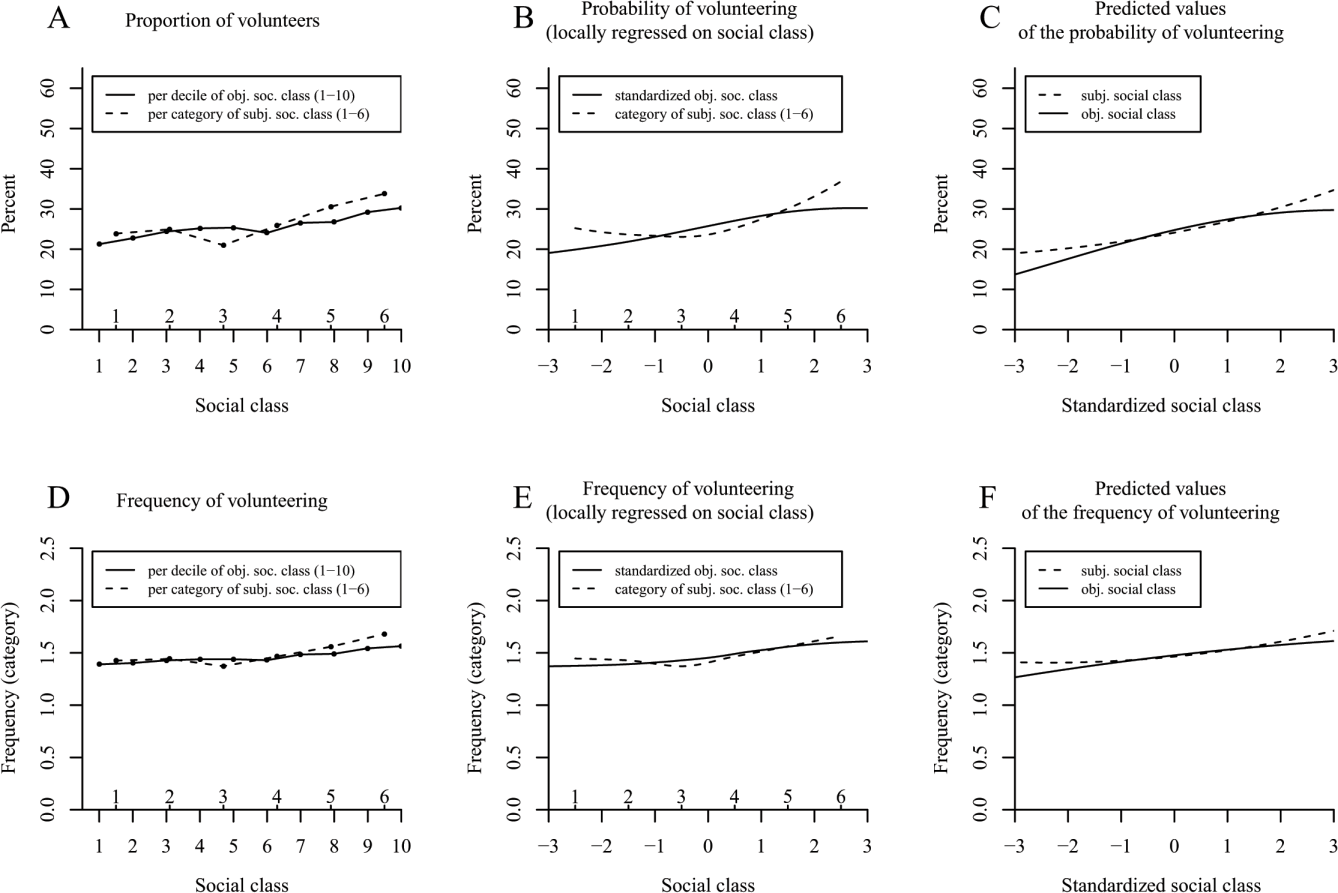
The positive effect of social class on volunteering in charitable activities using the complete data of the ISSP (Study 6). Panel A shows the proportion of volunteers per decile of objective social class (x-axis caption under the axis) and per category of subjective social class (x-axis caption above the axis). Panel B uses local likelihood fitting (Locfit) to adjust a curve to the raw data and illustrates the probability of volunteering by social class. Panel C shows the predicted values for the probability of volunteering determined via multilevel logistic regression. Panels D–F illustrate the frequency of volunteering using four categories (0 = no; 3 = yes, 6 or more times). Panel D shows the frequency of volunteering per decile of objective social class and per category of subjective social class. Panel E shows LOESS curves for the frequency of volunteering by social class. Panel F shows the predicted values for the frequency of volunteering determined via multilevel ordinary regression. Panels B–C and E–F distinguish between a curve for objective social class (solid line; x-axis caption under the axis; *N* = 37,136) and a curve for subjective social class (dashed line; 1 = lower class, 6 = upper class; *N* = 32,257).

Across all countries, the solid line in Fig 8A indicates a slight increase in the proportion of volunteers with increasing deciles of objective social class. This slope was also observable when we fit a Locfit curve to the raw data (Fig 8B, solid line). Table 6 (Model 1, column 1; see the solid line in Fig 8C for the predicted values) reveals that this linear effect was significant across all countries even though the slope was slightly attenuated with increasing objective social class. Without the covariates age and sex, the attenuation disappeared and changed into a clear cut linearity (objective social class: *OR* = 1.16, *z* = 6.13, *p* < .001; objective social class^2^: *OR* = 0.98, *z* = -1.62, *p* = .11).

**Table 6:** Study 6: Separate Multilevel Regressions of Volunteering (in Charitable Activities) on Subjective Social Class and Objective Social Class (with Data from the International Social Survey Program).

	Volunteering (yes/no)ª		Frequency of volunteering^b^ (Multilevel ordered probit model)		Frequency of volunteering^b^ (Multilevel ordinary regression model)	
	*OR*	*z*	*b*	*z*	*b*	*z*
**Model 1 (*N* = 37,136)**						
Objective social class	1.18	6.46***	.094	6.60***	.058	6.56***
Objective social class^2^	0.97	-2.50*	-.012	-2.14*	-.004	-1.05
Gender	1.12	4.69***	.089	6.38***	.065	7.13***
Age	1.01	10.26***	.005	12.91***	.004	14.51***
**Model 2 (*N* = 32,257)**						
Subjective social class	1.15	5.25***	.076	5.32***	.050	5.32***
Subjective social class^2^	1.01	0.90	.012	1.89	.011	2.74**
Gender	1.09	3.26**	.074	4.96***	.055	5.74***
Age	1.01	9.22***	.005	11.17***	.004	12.42***

For Model 1, 37,136 subjects were nested within 30 countries. For Model 2, 32,257 subjects were nested within 28 countries. Objective social class and subjective social class were standardized across all subjects separately for each country. *OR* = Odds Ratio. *b* = estimated coefficient of the multilevel ordered probit model. Gender: 1 = male; 2 = female.

^*a*^ Multilevel logistic model (0 = nonvolunteer; 1 = volunteer).

^b^ 0 = no; 3 = yes, 6 or more times.

* *p* < .05.

** *p* < .01.

*** *p* < .001 (two-tailed).

For the effect of objective social class on the frequency of volunteering, the solid lines in Fig 8D and 8E also point toward a slight positive effect. The corresponding multilevel analyses confirmed this positive effect. The ordered probit model furthermore revealed that this small positive effect was slightly attenuated with increasing social class (Table 6, Model 1, column 2), whereas the ordinary regression model indicated a positive linear effect (Table 6, Model 1, column 3; see the solid line in Fig 8F for the plotted predicted values). Without the covariates age and sex, once again, both statistical models indicated a positive linear effect without attenuation (multilevel ordered probit model: objective social class: *b* = .084, *z* = 6.29, *p* < .001; objective social class^2^: *b* = -.005, *z* = -0.94, *p* = .35; multilevel ordinary regression model: objective social class: *b* = .051, *z* = 6.06, *p* < .001; objective social class^2^: *b* = .001, *z* = 0.26, *p* = .80).

For subjective social class, the relation instead seemed to be more nonlinear. The proportion of volunteers first declined and reached its minimum for the category “lower middle class/upper working class.” Afterwards, the percentage of volunteers increased linearly (dashed line in Fig 8A). The LOESS analysis in Fig 8B (dashed line) also showed a u-shaped curve in the relation between the probability of volunteering and subjective social class. However, when computing a multilevel logistic regression to test the effect for significance, the quadratic effect of subjective social class did not reach significance in the model with the covariates (Table 6, Model 2, column 1; see the dashed line in Fig 8C for the predicted values) and also in the model without the covariates age and sex (subjective social class: *OR* = 1.13, *z* = 4.89, *p* < .001; subjective social class^2^: *OR* = 1.02, *z* = 1.17, *p* = .24). Thus, these models indicated a linear effect of subjective social class.

The same applied for the effect on the frequency of volunteering. Although the dashed lines in Fig 8D and 8E seemed to indicate a u-shaped relation, the multilevel ordered probit model revealed a positive linear relation (Table 6, Model 2, column 2). However, in the multilevel ordinary regression model (Table 6, Model 2, column 3; see the dashed line in Fig 8F for the plotted predicted values) and without the covariates age and sex, the results indeed indicated a quadratic relation (multilevel ordered probit model: subjective social class: *b* = .067, *z* = 4.83, *p* < .001; subjective social class^2^: *b* = .015, *z* = 2.27, *p* < .05; multilevel ordinary regression model: subjective social class: *b* = .044, *z* = 4.71, *p* < .001; subjective social class^2^: *b* = .012, *z* = 3.10, *p* < .01).

Contrary to Studies 4 and 5, older persons were more likely to volunteer and volunteered more often. Among the 30 countries that we investigated (or 28 countries for subjective social class), women were more likely to volunteer and volunteered more often.

**Fig 9:**
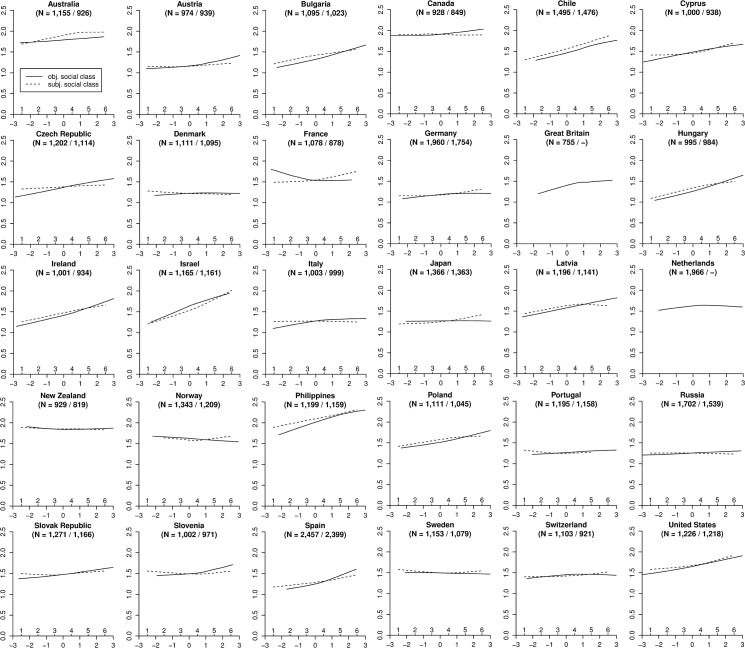
Effects of objective social class and subjective social class on volunteering in charitable activities separately for each country from the International Social Survey Program (ISSP; Study 6). The panels use local least squares fitting (LOESS; smoothing parameter = 1.0, polynomial = 1) to adjust a curve to the raw data and illustrate the frequency of volunteering (y-axis; four categories: 0 = no; 3 = yes, 6 or more times) by social class (x-axis). They distinguish between a curve for standardized objective social class (solid line; x-axis caption under the axis) and a curve for subjective social class (dashed line; x-axis caption above the axis; 1 = lower class, 6 = upper class). Sample sizes for each country are given in parentheses (*N*_*objective social class*_ / *N*_*subjective social class*_).

Not unexpectedly, the likelihood and frequency of volunteering varied between countries in all of our three models, χ²(1) ≥ 1986.1, *p*s < .001. Most interestingly, however, the effects of both the objective and the subjective social class measures varied significantly between countries, χ²(2) ≥ 31.9, *p*s < .001. To investigate these specific effects for each country, we conducted post hoc analyses on the frequency of volunteering separately for each country. First, comparable to Fig 8E, we fit locally weighted smoothing curves to the raw data for each country. Fig 9 shows the corresponding LOESS curves for the frequency of volunteering and illustrates how the relation varied between countries. We further conducted the corresponding ordered probit regression analyses separately for each country and separately for objective and subjective social class. These revealed a large range of significant relations. More precisely, we found significant curvilinear relations such as a slight u-shaped relation (France) and slight inverted u-shaped relations (Philippines, Great Britain, Latvia), nonsignificant relations (e.g., Denmark, New Zealand, Norway, Switzerland, Sweden), but mainly positive linear relations (e.g., Chile, Hungary, Ireland, Poland, Israel). What we did *no*t find, though, were the negative linear effects of social class on volunteering that would be expected on the basis of previous psychological research [1–3].

Altogether, Study 6 provided evidence for a moderating role of the observed country. In none of the countries, however, did we find a negative effect like the one indicated in the psychological literature [1–3]. Across all countries that were included in Study 6, we found a positive relation between social class and volunteering independent of whether objective or subjective social class was used as an independent variable.

## Study 7: Effect of Social Class on “Everyday Helping” (GSS)

Studies 1 to 3 examined how social class influenced donation behavior. In Studies 4 to 6, we extended these studies by examining how social class affected another important prosocial behavior: volunteering. However, critics might object that these two prosocial behaviors may be inappropriate for assessing prosociality in lower social class individuals and may have handicapped them in our studies so far. On the one hand, different tax incentives might contribute to the positive effect of social class on charitable giving [98,99]. On the other hand, volunteering involves organized help and requires individuals to contact volunteer organizations. Yet, lower social class individuals have less access to social institutions [17], and therefore, it may be more difficult for them to contact the necessary volunteer organizations. Hence, the primary objective of Study 7 was to broaden our focus of investigated prosocial behaviors to one behavior that might be more appropriate for persons in lower social realms and may better correspond to the reality of their lives. Thus, we used data from the American GSS and created a questionnaire that assessed “everyday helping” behaviors.

### Method

#### Participants

Study 7 used data from the GSS. Respondents answered several questions about their everyday helping behavior in 2002, 2004, and 2012. In addition, they provided information about their subjective social class, education, family income, and occupation in the same years (but there was no occupational data available for 2012). In total, 4,020 persons were asked about their everyday helping. A total of 103 persons did not provide information for all helping items, 15 persons did not provide the required demographic information, and a further 16 persons did not respond to the item asking about their subjective social class. Therefore, the final sample consisted of 3,902 persons (1,807 men; mean age = 46.78 years; *SD* = 17.33) for testing the effect of objective social class and a subsample of 3,886 persons for testing the effect of subjective social class.

#### Objective and subjective social class

We applied the same measures that we used in Studies 3 and 5. Participants reported a mean before-tax family income category of 16.75 (*SD* = 5.94) in 2012 and of 16.16 (*SD* = 5.41) in the other years, a mean educational category of 1.57 (*SD* = 1.19), a mean occupational prestige score of 43.56 (*SD* = 14.22), and a mean subjective social class category of 2.43 (*SD* = 0.67). The final score for objective social class ranged from -2.49 to 2.69. Subjective social class was significantly correlated with objective social class, *r* = .42, *p* < .001.

#### Everyday helping

Respondents were asked how often they had engaged in each of the following actions during the past 12 months: (a) given food or money to a homeless person, (b) returned money to a cashier after receiving too much change, (c) allowed a stranger to go ahead of them in line, (d) offered their seat on a bus or in a public place to a stranger who was standing, (e) looked after people’s plants, mail, or pets while they were away, (f) carried a stranger’s belongings, such as groceries, a suitcase, or shopping bag, (g) given directions to a stranger, or (h) let someone they did not know well borrow an item of some value such as dishes or tools (see also S1 File). Answers were provided by choosing from six categories: (0) not at all in the past year, (1) once in the past year, (2) at least two or three times in the past year, (3) once a month, (4) once a week, or (5) more than once a week. We conducted an exploratory factor analysis that suggested a one-factor solution (α = .70). In our sample, the scale values ranged from 0 to 38 (*M* = 10.18; *SD* = 5.37).

### Results and Discussion

**Fig 10:**
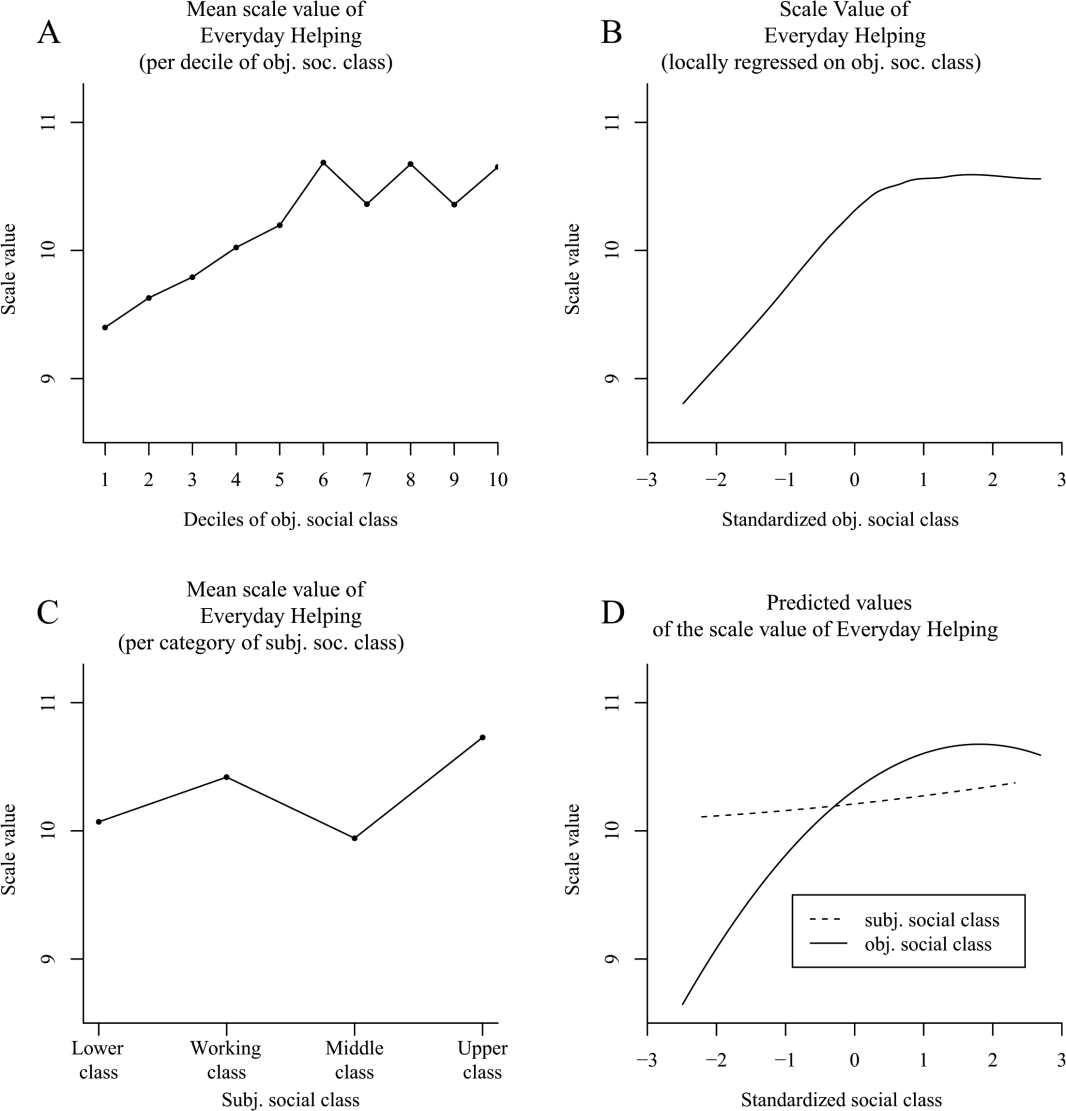
The positive effect of social class on everyday helping behavior in the American General Social Survey (Study 7). Panel A shows the mean scale value of the everyday helping scale per decile of objective social class. Panel B illustrates a LOESS curve (local least squares fitting) for the scale value of the everyday helping scale by standardized objective social class (*N* = 3,902). Panel C (*N* = 3,886 persons) shows the mean scale value of the everyday helping scale per category of subjective social class. Panel D illustrates the predicted scale values for the everyday helping scale determined via OLS regression. It distinguishes between a curve for subjective social class and a curve for objective social class.

Fig 10A shows an increase in the mean score of everyday helping with elevated deciles of social class up to the sixth decile. From that point on, the mean scale value increased and decreased with each subsequent decile. This tendency was also reflected in the applied local regression (Fig 10B). The curve rose until the end of the first half of the objective social class distribution and stayed straight in the second half. However, the regression analysis revealed a plain positive linear effect of objective social class on everyday helping (Table 7, Model 1; see the solid line in Fig 10D for the predicted values from the OLS regression). Yet this was most likely caused by the integration of the covariates age and sex in our analysis. When the analysis was conducted without those covariates, we found the graphically displayed positive effect of objective social class on everyday helping that attenuated with elevated social class (objective social class: *b* = .406, *t* = 4.74, *p* < .001; objective social class^2^: *b* = -.147, *t* = -2.06, *p* < .05).

**Table 7:** Study 7: Separate Regressions for Everyday Helping on Subjective Social Class and Objective Social Class (with Data from the American General Social Survey).

	Model 1 (*N* = 3,902)			Model 2 (*N* = 3,886)		
	*N*	*b*	*t*	*N*	*b*	*t*
Objective social class		.397	4.74***			
Objective social class^2^		-.110	-1.58			
Subjective social class					.058	0.67
Subjective social class^2^					.006	0.08
Gender		-.776	-4.63***		-.805	-4.78***
Age		-.061	-12.66***		-.062	-12.64***
Year						
2002	1,315			1,311		
2004	1,313	1.200	5.90***	1,309	1.213	5.94***
2012	1,274	.218	1.06	1,266	.238	1.16

Objective social class and subjective social class were standardized across all households per year. The reference value for year was 2002. *b* = unstandardized regression coefficients. Gender: 1 = male; 2 = female.

*** *p* < .001 (two-tailed).

For subjective social class, the curve in Fig 10C on the whole shows a straight line without increase or decrease. The regression analysis corroborated this impression by indicating no effect of subjective social class at all (Table 7, Model 2; see the dashed line in Fig 10D for the predicted values from the OLS regression). This nonrelation held when we executed the analysis without the covariates age and sex (subjective social class: *b* = -.093, *t* = -1.06, *p* = .29; subjective social class^2^: *b* = -.026, *t* = -0.39, *p* = .70).

Focusing on the mentioned covariates in both Models 1 and 2, we found that men and younger persons reported more everyday helping than did women and older people. In addition, there was variation in everyday helping across the observed years.

To sum up, Study 7 showed a positive effect of objective social class on everyday helping and no effect of subjective social class.

## Study 8: Effect of Social Class in the Trust Game (SOEP)

The goal of Study 8 was to extend our research focus to a prosocial behavior that could be observed directly: the allocation of points in an economic game. The question of whether elevated social class results in the allocation of more or fewer points in economic games has been investigated before and has produced rather inconsistent results [1,3,41,62]. In the dictator game, an economic game in which one player disposes of a certain resource (in this case: stickers) and is asked to give any amount he/she wants to another unknown player without the chance of getting anything back, Chinese children from higher income families allocated fewer stickers to other children than children from lower income families [3]. As opposed to this, British children from higher income families were reported to donate more stickers in the dictator game than children from lower income families [62]. Similar opposing results have been reported for the trust game, an economic game in which the second player has the opportunity to give back some of his/her resources to the first player (for additional methodological details about this economic game, see [100]). Lower social class individuals were found to be more trusting than higher class individuals in a US sample in a trust game in which participants had to allocate points to a stranger [1], whereas in a real-pay trust game, no class effects on trust were observed in a Dutch sample [41].

In Study 8, we reexamined the relation between social class and prosocial behavior in such an economic game. Fortunately and rather uncommon for a large panel, the SOEP also provides information on behavior in a real-pay trust game. Participants played a variant of the trust game [101] across 3 consecutive years. The trust game yields the advantage of simultaneously measuring trust and trustworthiness [100,102]. Both are indicators of prosociality because they activate the participant’s concern for others [103,104].

### Method

#### Participants

Study 8 used data from the German SOEP (Version 29). The trust game was administered in addition to the main survey to a randomly selected subgroup of 1,500 persons (750 Player 1; 750 Player 2) in the years 2003 to 2005. Only one member per household was allowed to participate in the trust game. Once assigned to be either Player 1 or Player 2, the respondents maintained this role in consecutive years. Of the 1,500 persons selected, 79 persons did not respond to the SOEP as a whole, specifically did not take part in the trust game, or did not provide any information about their social class in the main SOEP. Thus, the final sample included 1,421 persons (705 men; mean age = 50.34 years; *SD* = 17.14) who provided full information and took part in the trust game once to three times (mean = 2.69 times; making a total of 3,819 observations).

#### Procedure

In 2003, the main SOEP was conducted in the homes of the participants. After that, they were asked to participate in the trust game. The participants were told that they were assigned to another person and would receive money depending on their own and the other person’s choices in the game. Because the experimenters wanted to minimize dependencies in the data, participants were informed that they were assigned to different partners in 2004 and 2005.

#### Trust game

The participants played a trust game similar to the trust game used by Berg et al. [101] either as Player 1 or Player 2. Both Player 1 and Player 2 received 10 points as seed capital. They were told that they could keep the points for themselves or that they could fully or partially allocate some of their points to the other player. They were further instructed that (a) for each point they kept, they would receive one euro, (b) for each point they allocated to the other player, the other player would receive two euros, and (c) conversely, for each point the other player allocated to them, they would receive two euros themselves. To reduce bystander effects and to maintain the original double-blind design [101], participants were told to write down their decision on a form and put it in a sealed envelope, which was given to the interviewer [105]. Player 1 was informed that he/she would be the first to make his/her decision. He/she was further told that Player 2 would come to know his/her decision before he/she made his/her decision. In this game, the behavior of Player 1 is therefore interpreted as trust behavior. Player 2 was told how many points Player 1 allocated to him/her. He/she could subsequently decide how many of his/her 10 points he/she wanted to send to Player 1 in return. In the trust game, the behavior of Player 2 is therefore interpreted as trustworthiness (Although the game is set up as a trust game in the SOEP, some researchers would label the game a sequential dictator game [106]. However, even in this case, the allocation of points to the other player would still be a prosocial act.)

But although participants were told that they were assigned to another participant, they actually played with a fictional partner. This procedure was necessary because of the requirements of representative sampling in a large panel. This led to some special demands on the implementation of the game. Interviewers surveyed either only participants from the first group (i.e., Player 1) or only from the second group (i.e., Player 2) to prevent both the interviewers and participants from knowing that the partners were fictional. Because Player 2 received points from a fictional player, a pretest was conducted with another sample. According to the distribution of the numbers of points sent on this pretest, a certain number of points were randomly allocated to Player 2. In our sample, Player 1 sent on average 5.40 points (*SD* = 2.57). Player 2 received on average 5.15 points (*SD* = 2.79) and sent 4.90 points (*SD* = 2.68) himself/herself. After Player 2’s decision, his/her payoff could easily be calculated. To determine the payoff for Player 1, he/she was alphabetically matched with one of the players in the second group. All participants received their individual payoff (*M* = 14.97€) together with a letter of thanks by mail.

#### Objective social class

A measure of objective social class was generated in the same way as in Study 4. Participants reported a mean weighted household after-tax income category of 3.68 (*SD* = 1.30), a mean educational category of 3.51 (*SD* = 1.32), and a mean occupational prestige score of 42.63 (*SD* = 12.49). The final score for objective social class ranged from -2.24 to 2.86.

#### Analytical procedure

Similar to Study 4, we faced a challenge from respondents playing the trust game in consecutive years. Thus, we applied multilevel ordinary regression models with repeated measurements nested within persons. Because demographic variables might affect behavior in the trust game [41,107,108], we controlled for age and gender in our regression analyses. For Player 2, we further controlled for the number of points received in each analysis [109].

### Results and Discussion

**Fig 11:**
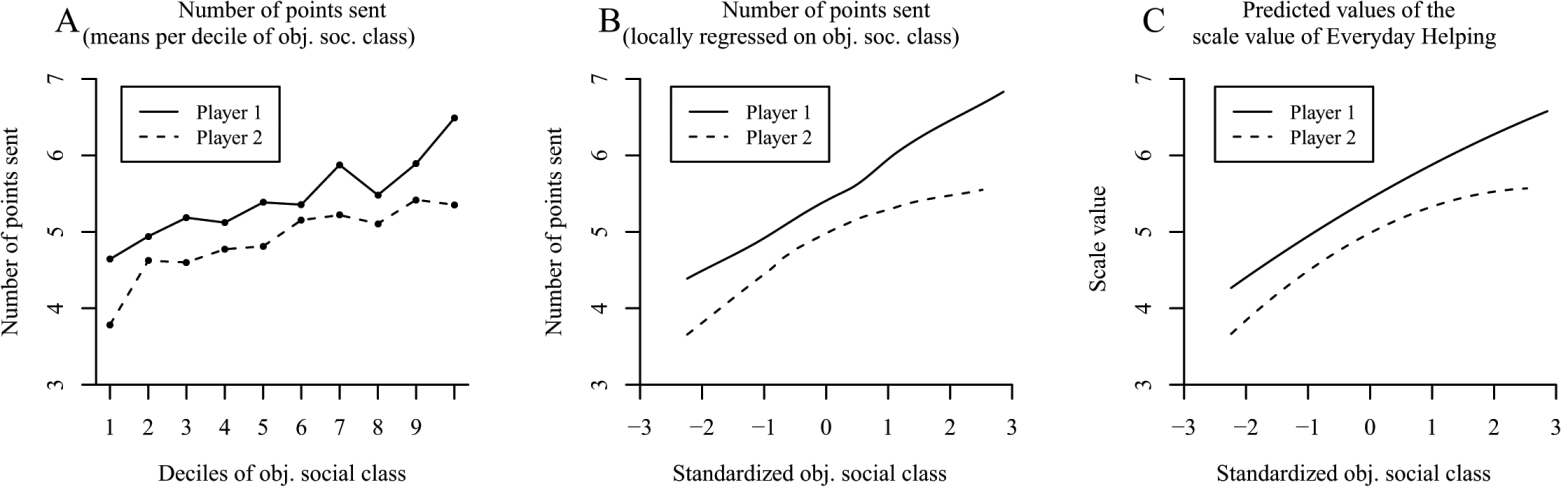
The positive effect of social class on the number of points sent in a trust game in the German SOEP (Study 8). Panel A illustrates the mean number of points sent per decile of objective social class. Panel B shows LOESS curves (local least squares fitting) for the number of points sent by objective social class. Panel C shows the predicted values for the number of points sent determined via multilevel ordinary regression. Panels A–C distinguish between Player 1 (with 1,901 observations) and Player 2 (with 1,918 observations). For Player 2, points sent were controlled for points received.

First, we determined the mean number of points sent per decile of social class for Player 1 (solid line in Fig 11A) and Player 2 (dashed line in Fig 11A). This first impression indicated an increase in the number of points sent with elevated social class. Next, we again adjusted LOESS curves to the raw data. Fig 11B points toward a distinctive increase in points sent with elevated social class for Player 1 (solid line) and Player 2 (dashed line).

As can be seen in Table 8, for both players, we were also able to statistically confirm the increase in the number of points sent with elevated social class in the applied multilevel ordinary regression models (see Fig 11C for a plot of the predicted values). Moreover, the effect held when we left out the covariates age and sex in our calculations for Player 1 (objective social class: *b* = .443, *z* = 5.18, *p* < .001; objective social class^2^: *b* = -.026, *z* = -0.37, *p* = .71) and for Player 2 (objective social class: *b* = .403, *z* = 5.32, *p* < .001; objective social class^2^: *b* = -.073, *z* = -1.15, *p* = .25). Both for Player 1, *ICC* = .407, χ²(1) = 261.3, *p* < .001, and Player 2, *ICC* = .236, χ²(1) = 88.3, *p* < .001, there was some stability in the number of points sent across the three years.

**Table 8:** Study 8: Multilevel Ordinary Regression Models for Testing the Effect of Social Class on Points Sent in the Trust Game (with Data from the German SOEP).

	Player 1			Player 2		
	*N*	*b*	*z*	*N*	*b*	*z*
Objective social class		.468	5.50***		.421	5.54***
Objective social class^2^		-.024	-0.35		-.075	-1.20
Gender		.207	1.37		.325	2.46*
Age		-.016	-3.70***		.005	1.28
Points received					.390	20.99***
Year						
2003	657			655		
2004	650	.350	3.23**	655	-.064	-0.55
2005	594	.579	5.16***	608	.057	0.48

For Player 1, 1,901 observations were nested within 709 persons. For Player 2, 1,918 observations were nested within 712 persons. Objective social class was standardized per year across all subjects. The reference value for year was 2003. *b* = unstandardized regression coefficients. Gender: 1 = male; 2 = female.

* *p* < .05.

** *p* < .01.

*** *p* < .001 (two-tailed).

Age was a negative predictor of points sent by Player 1—indicating that older persons sent fewer points in the trust game when they were Player 1—but not for points sent by Player 2. By contrast, gender predicted points sent only for Player 2. When they were Player 2, women sent more points than men.

In summary, participants in higher social classes allocated more points to an assigned stranger in a trust game than participants in lower social classes. The effect was independent of participants’ age, sex, or the number of points received in the game—ruling out mere return service as a possible explanation.

## General Discussion

Whether or not higher social class individuals act in more generous and helpful ways than lower social class individuals is an important question of modern society. But whereas research outside the field of psychology—despite some heterogeneity—has primarily found a u-shaped or a positive relation between social class and prosocial behavior, in the psychological literature, the perspective on social class is rather negative. According to this literature, higher social class individuals are less prosocial than their lower social class counterparts [1–5].

We aimed to thoroughly analyze the proposed negative effect and were inspired by other large-scale tests that reexamined popular scientific findings by using large, representative, international, and publicly available datasets that were professionally administered by large research organizations ([41,110,111]; for the data and the syntax we used for the statistical analyses, see S2 File). However, in contrast to previous results in the psychological field, none of our own analyses with large representative panels (with up to 37,136 participants) replicated the proposed negative effect. Across eight studies, we predominantly found a positive effect of social class on various forms of prosociality. Compared with lower social class individuals, higher social class individuals were more likely to make any charitable donation and gave a higher percentage of their family income to charity (Studies 1–3), were more likely to volunteer and volunteered more often (Studies 4–6), were more helpful in everyday interactions (Study 7), and were more trusting and trustworthy when interacting with a stranger in a trust game (Study 8). Furthermore, as our supplementary analyses showed (see S3–S12 Tables), this positive effect was almost always equally driven by each indicator of social class—income, education, and occupational prestige.

In Study 1, we found the previously posited u-shaped curve when regressing the relative amount of money donated on social class in donor households [36,40,42–45]. Yet, when we conducted the same analysis using donor and nondonor households together and therefore accounted for the fact that a higher percentage of lower social class households do not make any charitable contributions at all, the u curve changed into a positive linear increase [43,44]. Similarly, in Study 2, a predominantly decreasing curve in donor households transformed into an increasing curve when nondonor households were entered into a joint analysis. Moreover, this positive effect was also found when we conducted a combined analysis of donation behavior that integrated calculations on both the probability of donating anything at all and the relative monetary amounts of the donations.

Furthermore, in Studies 1, 3, 4, and 6, we observed a curvilinear relation between social class and prosociality such that the linear positive slope attenuated with increasing social class. However, this does not minimize the importance and dominance of the positive effect in any way. Even after accounting for the attenuated slope, higher social class individuals still had the highest probability and frequency of making charitable donations (Studies 1 and 3) and were more likely to volunteer (Studies 4 and 6).

With regard to a possible moderating effect of the observed country (United States, Germany, or some other 28 countries) and the measurement of social class (conceptualized either as a composition of socioeconomic indicators or as a subjective appraisal of one’s own social class rank), we found inconsistent results. Whereas country did not moderate the effect of social class on donating (Studies 1–3) or on volunteering in the German and US samples (Studies 4–5), there was considerable variation in the effect of social class on volunteering in other countries (Study 6). In addition, a different measure of social class did not change the direction of the effects on donating (Study 3) or volunteering (Studies 5–6). However, for everyday helping, there was a positive effect of objective social class and no effect of subjective social class. Thus, although there was some variation in effects due to the inclusion of moderators, they did not work in favor of a negative effect. That is, under *no* condition in any study did we find the negative effect that would have been expected on the basis of previous results in the psychological field [1–3].

### Limitations, Challenges, and Future Directions

We believe that this research embodies some potentially advantageous characteristics: First, we examined large and representative samples (1,421 < *N* < 37,136), whereas most previous studies have based their analyses on multiple small samples that sometimes even consisted entirely of students. This reduced not only the statistical power of each single study but also the power of the combination of these studies. That is, when combining multiple studies in one article, the persuasiveness and robustness seem to increase, but actually, the power diminishes as more statistical tests are executed [48–53,56–58]. This problem has recently been discussed extensively in the literature and has led to an increase in caution against the results of these small-sample multistudy articles [51] that are best avoided [48]. By contrast, our large samples allowed for an analysis of the proposed negative effect with extremely high statistical power.

Second, regarding the measurement of social class, we used a composite measure that comprised the three objective socioeconomic indicators of income, education, and occupational prestige in our analyses (we were restricted to income and educational attainment only in Study 2 and for the year 2012 in the studies using the GSS; see [17,69–71]). By using the same state-of-the-art composite measure in all of our analyses, we minimized the possible dominating effect of any single indicator. Furthermore, we complemented our main analyses by also using a measure of subjective social class.

Third, we were cautious when we choose and handled the covariates. We always used the same covariates—age and gender—across all studies (because Studies 1 and 2 analyzed donation behavior at the household level, age and gender were not included as covariates in these analyses), whereas previous research often used covariates (age, ethnicity, religiosity, and different combinations of them) that varied inconsistently across studies. Furthermore, following Simmons et al.’s [58] advice, we additionally reported the results of all analyses without the inclusion of covariates to provide evidence for the robustness of the results across different analytical methods.

Despite these potential positive features of our research, there are clearly some limitations that should be noted. First, because we relied on representative international survey panels, seven of our eight studies were based on self-reports. In the studies using the ISSP and the GSS, respondents retrospectively reported how often they donated and volunteered for charity and how often they had engaged in some prosocial everyday behaviors in the last year. In the study using the CEX, participants documented their income and expenses (also those for charity) in a “Diary Survey”—and we were therefore able to at least minimize self-serving hindsight bias [112]. Yet, all these self-reports are necessarily subject to social desirability and self-presentation biases [113,114], and these biases might even be enhanced among higher social class individuals [115–117]. Importantly, these potential limitations do not apply to the results of our Study 8: Fortunately and rather uncommon for a large panel, the SOEP also assesses directly observed prosocial behavior: the allocation of points in an economic game. Specifically, participants played a variant of the “trust game” [101] across 3 consecutive years, and we simultaneously measured trust and trustworthiness [100,102].

Second, we were able to use only a limited selection of prosocial behaviors. Prosociality is a broad concept that covers various actions that are aimed at benefitting others [59–61]. In the present studies, we assessed four very different behaviors—from the allocation of money or money-like points to face-to-face helping in an organized or everyday setting. Future research, however, should expand the focus to other forms of prosociality (e.g., to observable helping behaviors in field experiments using non-student populations).

A third limitation lies in the unclear personal motivation for prosociality. That is, individuals might act generously not just because they want to help others. It is also possible that they act benevolently because they expect some sort of direct or indirect reciprocity that may help them in the future [118–121] or because of a desire for “prosocial” prestige [34,122,123]. Moreover, the (expected) prosocial behavior of others may have established a social norm that prompted individuals to behave philanthropically when they would not have done so in private [124–126]. For example, in a field experiment, Martin and Randal [127] placed a transparent donation box in an art gallery with free admission and found varying frequencies and amounts of donations depending on whether or not, how much, and what kind of money (coins vs. bills) was visible in the box. Thus, even if the motivation for the prosocial act is not relevant in terms of the key question of the present paper, future research should try to uncover the underlying motivation for prosociality and examine whether individuals from different social classes vary in their motivation for prosociality. A recent study by Stephens et al. [128] at least pointed in the direction of different motives: Students whose parents did not have a college degree (and who therefore might be from a lower social class) endorsed more interdependent motives (e.g., helping their families, giving back to their communities) for attending a university than students who had at least one parent with a college degree.

In sum, although our results certainly do not fit with those presented in the psychological literature [1–3], they are in line with other research on the effects of social class on prosociality. For example, in research outside the field of psychology involving large-scale investigations in Taiwan, Canada, and the United States, individuals of the higher social realms were reported to volunteer and donate more often and to donate more to charity than those in the lower realms [8–11,37–40]. In terms of the monetary amounts of the donations relative to household income, the literature in sociology and economics furthermore reported a u-shaped relation to a household’s social class [36,40,42–45]. However, this held only for donor households. When donor and nondonor households were combined in a joint analysis, as also corroborated in our Studies 1 and 2, the artifactual u curve (or decreasing tendency as we found in Study 2) changed into a positive effect ([43,44]; for some other methodological issues that may produce the u curve, see also [36]).

All in all, cumulative evidence therefore strongly points toward a positive effect rather than a negative effect of social class on prosocial behavior.

### Implications for the Theories on Social Class and on Prosociality

With that said, it is also worthwhile to speculate about the implications for the posited social-cognitive perspective on social class [4]. Psychological research, compared with research in sociology and economics, has only recently begun to focus on the effects of social class on cognition and behavior [70]. At this point in time, the outcome has been rather negative—higher social class individuals are supposed to favor dispositional explanations for their fate [19], to exhibit reduced empathy [14,16], and to be less friendly and more concerned with themselves [22]. Finally, because they are less involved in and less worried about their environment, some researchers have suggested that they have developed a social-cognitive orientation toward unsociality, unethicality, utilitarism, and less compassion (for the detailed theory, see [4]).

We would not go as far as to querying all subparts of this posited social-cognitive perspective. For example, increased feelings of independency and a stronger sense of control among higher social class individuals have been found by many researchers [17–21] and were also replicated in large samples [41]. Yet, our research showed that at least one of the fundamental components of this perspective, the prediction of unsocial behavior, may be fragile. But our results are not the only ones to raise concerns with regard to the sophistication of the perspective. According to the social-cognitive perspective and important with regard to its theory building, research has indicated that higher social class individuals act less ethically [26–30]. However, this negativistic view on high social class has been undermined by studies with representative samples [41] and by power considerations [53]. Taking into account this additional flaw, we are not able to avoid questioning the generalizability and conclusiveness of this social-cognitive perspective on social class.

But our results also have important implications for a new and highly elaborated theory on the development of prosociality. In this theory, Keltner et al. [5] present both a bunch of sociocultural mechanisms that influence prosocial behavior and their underlying genetic and neurophysiological processes. As a prime example of their theory, the negative effect of social class on prosociality is discussed in depth, and the potential mechanisms that should influence prosocial behavior are enumerated from A to Z by using this example ([5], p. 449–450). However, as is now known from the present research, this example may not be conclusive at all, leading to an enhanced and certainly unintended fragility of the theory of prosociality.

### A Few Final Thoughts on Implications for Psychological Research

Our present research showed that one highly published and frequently cited finding from psychological research, the proposed negative effect of social class on prosociality, may not be as robust as expected. But it also showed that the intensive cross-testing of theories via direct and conceptual replications might be crucial for the future and reputation of psychological research [129]. As Schmidt [13] put it, “Replication is one of the most important tools for the verification of facts within the empirical sciences” (p. 90; see also [130]). It even helps to advance our theories by showing boundary conditions or moderators that helped to produce the effects that were found in the first place and, thus, provides starting points for future research ([12,13,131,132]; for some recent concrete examples, e.g., see Donnellan, Lucas, & Cesario [133], on the results of Bargh & Shalev, [134]; or Galak, LeBoeuf, Nelson, & Simmons, [135], and Wagenmakers, Wetzels, Borsboom, & van der Maas, [136], on the results of Bem, [137]; see also the first results of the “many labs” replication project, [138]).

To conclude, theory and empirical results are inextricably interwoven with each other (for a recent discussion, see, e.g., [139]). Studies are guided by theory, and theory should be informed by studies. Our studies constitute one tessera (perhaps even eight tesseras) that might not be easily integrated into the elaborated mosaic of negative effects of higher social class. Future work on both the theoretical and empirical sides of this topic is needed to build a more conclusive and consistent body of evidence that will sufficiently address this important topic—independent of what one would like to be true or what one thinks is true [140].

## Supporting Information

S1 File Additional Information on the Development of the Everyday Helping Scale. (TXT)

S2 File Data and Syntax of the Present Analyses as well as the Calculations of Statistical Power. Files are provided separately for each study. For more information, see also the included txt-file.
(ZIP)

S1 Table Study 2: Effects of Social Class and its Quadratic Term on Donations to Charities, Educational Institutions, Religious Organizations, and Political Parties (with Data from the American CEX). Objective social class was standardized across all households. *OR* = odds ratio; *b* = unstandardized regression coefficient. ^*a*^ Logistic Model (0 = nondonor; 1 = donor). ^b^ Nonlinear ordinary regression model computed excluding nondonors. ^c^ Nonlinear regression model including donor and nondonor households. *** *p* < .001 (two-tailed).
(DOCX)

S2 Table Study 2: Overall Effects (Determined via Tobit Regression) of Social Class and its Quadratic Term on Donations to Charities, Educational Institutions, Religious Organizations, and Political Parties (with Data from the American CEX). Objective social class was standardized across all households. ** *p* < .01. *** *p* < .001 (two-tailed).
(DOCX)

S3 Table Study 1: Separate Regressions of Donating on Social Class, Income, Education, Job Prestige, and their Quadratic Terms (with Data from the German Socio-Economic Panel). Predictor variables were standardized across all households. *OR* = odds ratio; *b* = unstandardized regression coefficient. ^*a*^ Logistic Model (0 = nondonor; 1 = donor). ^b^ Nonlinear ordinary regression model computed excluding nondonors. ^c^ Nonlinear regression model including donor and nondonor households. * *p* < .05. ** *p* < .01. *** *p* < .001 (two-tailed).
(DOCX)

S4 Table Study 1: Separate Tobit Regressions of Donating on Social Class, Income, Education, Job Prestige, and their Quadratic Terms (with Data from the German Socio-Economic Panel). Predictor variables were standardized across all households. ** *p* < .01. *** *p* < .001 (two-tailed).
(DOCX)

S5 Table Study 2: Separate Regressions of Donating on Social Class, Income, Education, and their Quadratic Terms (with Data from the American CEX). Predictor variables were standardized across all households. *OR* = odds ratio; *b* = unstandardized regression coefficient. ^*a*^ Logistic Model (0 = nondonor; 1 = donor). ^b^ Nonlinear ordinary regression model computed excluding nondonors. ^c^ Nonlinear regression model including donor and nondonor households. ** *p* < .01. *** *p* < .001 (two-tailed).
(DOCX)

S6 Table Study 2: Separate Tobit Regressions of Donating on Social Class, Income, Education, and their Quadratic Terms (with Data from the American CEX). Predictor variables were standardized across all households. *** *p* < .001 (two-tailed).
(DOCX)

S7 Table Study 3: Separate Regressions of Donating on Social Class, Income, Education, Job Prestige, and their Quadratic Terms (with Data from the American GSS). Predictor variables were standardized across all subjects separately for each year. Model 1 was computed including the covariates age and sex. Model 2 was computed without covariates. Sample sizes were different for each predictor variable (objective social class: *N* = 3,975; income: *N* = 3,536; educational status: *N* = 3,974; job prestige: *N* = 2,547). *OR* = odds ratio. *b* = estimated coefficient of the ordered probit model. ^*a*^ Logistic regresison (0 = nondonor; 1 = donor). ^b^ 0 = not at all in the past year; 5 = more than once a week. * *p* < .05. ** *p* < .01. *** *p* < .001 (two-tailed).
(DOCX)

S8 Table Study 4: Separate Multilevel Regressions for Volunteering on Social Class, Income, Education, Job Prestige, and their Quadratic Terms (with Data from the German SOEP). Predictor variables were standardized per year across all subjects. Model 1 was computed including the covariates age and sex. Model 2 was computed without covariates. Sample sizes (observations) were different for each predictor variable (objective social class: *N* = 82,966; income: *N* = 74,053; educational status: *N* = 79,663; job prestige: *N* = 46,327). Observations were nested within persons. *OR* = odds ratio. *b* = estimated coefficient of the multilevel ordered probit model. ^*a*^ Multilevel logistic regression model (0 = nonvolunteer; 1 = volunteer). ^b^ 0 = never, 3 = every week. ** *p* < .01. *** *p* < .001 (two-tailed).
(DOCX)

S9 Table Study 5: Separate Regressions of Volunteering on Social Class, Income, Education, Job Prestige, and their Quadratic Terms (with Data from the American GSS). Predictor variables were standardized across all subjects separately for each year. Model 1 was computed including the covariates age and sex. Model 2 was computed without covariates. Sample sizes were different for each predictor variable (objective social class: *N* = 3,983; income: *N* = 3,540; educational status: *N* = 3,982; job prestige: *N* = 2,551). *OR* = odds ratio. *b* = estimated coefficient of the ordered probit model. ^*a*^ Logistic regression (0 = nondonor; 1 = donor). ^b^ 0 = not at all in the past year; 5 = more than once a week. * *p* < .05. ** *p* < .01. *** *p* < .001 (two-tailed).
(DOCX)

S10 Table Study 6: Separate Multilevel Regressions of Volunteering on Social Class, Income, Education, Job Prestige, and their Quadratic Terms (with Data from the ISSP). Predictor variables were standardized across all subjects separately for each country. Model 1 was computed including the covariates age and sex. Model 2 was computed without covariates. Sample sizes were different for each predictor variable (objective social class: *N* = 37,136; income: *N* = 25,622; educational status: *N* = 36,695; job prestige: *N* = 22,764). Subjects were nested within countries. *OR* = Odds Ratio. *b* = estimated coefficient of the multilevel ordered probit model. ^*a*^ Multilevel logistic model (0 = nonvolunteer; 1 = volunteer). ^b^ 0 = no; 3 = yes, 6 or more times. ** *p* < .01. *** *p* < .001 (two-tailed).
(DOCX)

S11 Table Study 7: Separate Regressions of Everyday Helping on Social Class, Income, Education, Job Prestige, and their Quadratic Terms (with Data from the American GSS). Predictor variables were standardized across all subjects separately for each year. Model 1 was computed including the covariates age and sex. Model 2 was computed without covariates. Sample sizes were different for each predictor variable (objective social class: *N* = 3,902; income: *N* = 3,486; educational status: *N* = 3,901; job prestige: *N* = 2,496). *b* = unstandardized regression coefficients. * *p* < .05. ** *p* < .01. *** *p* < .001 (two-tailed).
(DOCX)

S12 Table Study 8: Separate Multilevel Ordinary Regression Models for Testing the Effects of Social Class, Income, Education, Job Prestige, and their Quadratic Terms on Points Sent in the Trust Game (with Data from the German SOEP). Predictor variables were standardized per year across all subjects. Model 1 was computed including the covariates age and sex. Model 2 was computed without covariates. Sample sizes (observations) were different for each predictor variable and player (objective social class: *N* = 1,901/1,918; income: *N* = 1,785/1,809; educational status: *N* = 1,842/1,881; job prestige: *N* = 946/1,031). Observations were nested within persons. *b* = unstandardized regression coefficients. * *p* < .05. *** *p* < .001 (two-tailed).
(DOCX)
