# Supplementary material for: A Large Scale Test of the Effect of Social Class on Prosocial Behavior
Source: PLoS One. 2015 Jul 20;10(7):e0133193. doi: 10.1371/journal.pone.0133193 (PMC4507988; doi:10.1371/journal.pone.0133193)
Supplement: S1 Table — Objective social class was standardized across all households. OR = odds ratio; b = unstandardized regression coefficient. a Logistic Model (0 = nondonor; 1 = donor). b Nonlinear ordinary regression model computed excluding nondonors. c Nonlinear regression model including donor and nondonor households. *** p < .001 (two-tailed). (DOCX) [file pone.0133193.s003.docx]

**Table S1. Study 2: Effects of Social Class and its Quadratic Term on Donations to Charities, Educational Institutions, Religious Organizations, and Political Parties (with Data from the American CEX)**

|  | **Donation (yes/no)ª** | | | **Relative monetary amounts of donations for donor households only^b^** | | | **Relative monetary amounts of donations for all households^c^** | | |
| --- | --- | --- | --- | --- | --- | --- | --- | --- | --- |
|  | ***N*** | ***OR*** | ***z*** | ***N*** | ***b*** | ***t*** | ***N*** | ***b*** | ***t*** |
|  | 32,052 |  |  | 21,303 |  |  | 32,052 |  |  |
| Objective social class |  | 1.83 | 45.48*** |  | -.724 | -16.29*** |  | -.048 | -1.64 |
| Objective social class² |  | 1.00 | 0.35 |  | .173 | 4.44*** |  | -.012 | -0.45 |

Objective social class was standardized across all households. *OR* = odds ratio; *b* = unstandardized regression coefficient.

*^a^* Logistic Model (0 = nondonor; 1 = donor). ^b^ Nonlinear ordinary regression model computed excluding nondonors. ^c^ Nonlinear regression model including donor and nondonor households.

*** *p* < .001 (two-tailed).
