# Supplementary material for: A Large Scale Test of the Effect of Social Class on Prosocial Behavior
Source: PLoS One. 2015 Jul 20;10(7):e0133193. doi: 10.1371/journal.pone.0133193 (PMC4507988; doi:10.1371/journal.pone.0133193)
Supplement: S2 Table — Objective social class was standardized across all households. ** p < .01. *** p < .001 (two-tailed). (DOCX) [file pone.0133193.s004.docx]

**Table S2. Study 2: Overall Effects (Determined via Tobit Regression) of Social Class and its Quadratic Term on Donations to Charities, Educational Institutions, Religious Organizations, and Political Parties (with Data from the American CEX)**

|  | ***N*** | ***Coeff.*** | ***t*** |
| --- | --- | --- | --- |
|  | 32,052 |  |  |
| Objective social class |  | .732 | 17.65*** |
| Objective social class² |  | -.124 | -3.37** |

Objective social class was standardized across all households

** *p* < .01. *** *p* < .001 (two-tailed).
