# Supplementary material for: A Large Scale Test of the Effect of Social Class on Prosocial Behavior
Source: PLoS One. 2015 Jul 20;10(7):e0133193. doi: 10.1371/journal.pone.0133193 (PMC4507988; doi:10.1371/journal.pone.0133193)
Supplement: S3 Table — Predictor variables were standardized across all households. OR = odds ratio; b = unstandardized regression coefficient. a Logistic Model (0 = nondonor; 1 = donor). b Nonlinear ordinary regression model computed excluding nondonors. c Nonlinear regression model including donor and nondonor households. * p < .05. ** p < .01. *** p < .001 (two-tailed). (DOCX) [file pone.0133193.s005.docx]

**Table S3. Study 1: Separate Regressions of Donating on Social Class, Income, Education, Job Prestige, and their Quadratic Terms (with Data from the German Socio-Economic Panel)**

|  | **Donation (yes/no)ª** | | | **Relative monetary amounts of donations for donor households only^b^** | | | **Relative monetary amounts of donations for all households^c^** | | |
| --- | --- | --- | --- | --- | --- | --- | --- | --- | --- |
|  | ***N*** | ***OR*** | ***z*** | ***N*** | ***b*** | ***t*** | ***N*** | ***b*** | ***t*** |
| Objective social class | 9,363 | 2.07 | 29.01*** | 4,907 | .005 | 0.18 | 9,260 | .158 | 11.47*** |
| Objective social class² |  | 0.97 | -1.21 |  | .133 | 6.08*** |  | .073 | 6.39*** |
| Income | 9,316 | 1.87 | 26.33*** | 4,907 | -.051 | -1.83 | 9,239 | .112 | 8.39*** |
| Income² |  | 0.95 | -3.02** |  | .102 | 5.68*** |  | .043 | 4.45*** |
| Educational status | 9,220 | 1.63 | 19.14*** | 4,851 | .030 | 0.99 | 9,120 | .122 | 8.02*** |
| Educational status² |  | 1.02 | 0.61 |  | .156 | 4.50*** |  | .083 | 4.80*** |
| Job prestige | 5,425 | 1.66 | 16.98*** | 2,924 | .141 | 5.76*** | 5,378 | .149 | 11.28*** |
| Job prestige² |  | 1.06 | 2.44* |  | .008 | 0.45 |  | .025 | 2.38* |

Predictor variables were standardized across all households. *OR* = odds ratio; *b* = unstandardized regression coefficient.

*^a^* Logistic Model (0 = nondonor; 1 = donor). ^b^ Nonlinear ordinary regression model computed excluding nondonors. ^c^ Nonlinear regression model including donor and nondonor households.

* *p* < .05. ** *p* < .01. *** *p* < .001 (two-tailed).
