# Supplementary material for: A Large Scale Test of the Effect of Social Class on Prosocial Behavior
Source: PLoS One. 2015 Jul 20;10(7):e0133193. doi: 10.1371/journal.pone.0133193 (PMC4507988; doi:10.1371/journal.pone.0133193)
Supplement: S4 Table — Predictor variables were standardized across all households. ** p < .01. *** p < .001 (two-tailed). (DOCX) [file pone.0133193.s006.docx]

**Table S4. Study 1: Separate Tobit Regressions of Donating on Social Class, Income, Education, Job Prestige, and their Quadratic Terms (with Data from the German Socio-Economic Panel)**

|  | ***N*** | ***Coeff.*** | ***t*** |
| --- | --- | --- | --- |
| Objective social class | 9,260 | .551 | 22.16*** |
| Objective social class² |  | .011 | 0.56 |
| Income | 9,239 | .452 | 18.98*** |
| Income² |  | -.014 | -0.84 |
| Educational status | 9,120 | .384 | 14.58*** |
| Educational status² |  | .084 | 2.83** |
| Job prestige | 5,378 | .361 | 16.24*** |
| Job prestige² |  | .019 | 1.09 |

Predictor variables were standardized across all households.

** *p* < .01. *** *p* < .001 (two-tailed)
