# Supplementary material for: A Large Scale Test of the Effect of Social Class on Prosocial Behavior
Source: PLoS One. 2015 Jul 20;10(7):e0133193. doi: 10.1371/journal.pone.0133193 (PMC4507988; doi:10.1371/journal.pone.0133193)
Supplement: S5 Table — Predictor variables were standardized across all households. OR = odds ratio; b = unstandardized regression coefficient. a Logistic Model (0 = nondonor; 1 = donor). b Nonlinear ordinary regression model computed excluding nondonors. c Nonlinear regression model including donor and nondonor households. ** p < .01. *** p < .001 (two-tailed). (DOCX) [file pone.0133193.s007.docx]

**Table S5. Study 2: Separate Regressions of Donating on Social Class, Income, Education, and their Quadratic Terms (with Data from the American CEX)**

|  | **Donation (yes/no)ª** | | | | **Relative monetary amounts of donations for donor households only^b^** | | | **Relative monetary amounts of donations for all households^c^** | | | |
| --- | --- | --- | --- | --- | --- | --- | --- | --- | --- | --- | --- |
|  | ***N*** | ***OR*** | | ***z*** | ***N*** | ***b*** | ***t*** | ***N*** | ***b*** | ***t*** | |
| Objective social class | 32,090 | 1.99 | 53.50*** | | 14,085 | -.228 | -6.95*** | 32,090 | .078 | | 6.37*** |
| Objective social class² |  | 0.94 | -4.94*** | |  | .135 | 4.98*** |  | .020 | | 1.79 |
| Income | 32,090 | 1.86 | 50.07*** | | 14,085 | -.608 | -20.34*** | 32,090 | -.039 | | -3.22** |
| Income² |  | 0.99 | -0.73 | |  | .478 | 17.65*** |  | .127 | | 10.97*** |
| Educational status | 31,515 | 1.70 | 42.58*** | | 13,916 | .141 | 4.62*** | 31,515 | .180 | | 14.88*** |
| Educational status² |  | 0.97 | -2.83** | |  | .096 | 3.61*** |  | .059 | | 5.29*** |

Predictor variables were standardized across all households. *OR* = odds ratio; *b* = unstandardized regression coefficient.

*^a^* Logistic Model (0 = nondonor; 1 = donor). ^b^ Nonlinear ordinary regression model computed excluding nondonors. ^c^ Nonlinear regression model including donor and nondonor households.

** *p* < .01. *** *p* < .001 (two-tailed).
