# Supplementary material for: A Large Scale Test of the Effect of Social Class on Prosocial Behavior
Source: PLoS One. 2015 Jul 20;10(7):e0133193. doi: 10.1371/journal.pone.0133193 (PMC4507988; doi:10.1371/journal.pone.0133193)
Supplement: S6 Table — Predictor variables were standardized across all households. *** p < .001 (two-tailed). (DOCX) [file pone.0133193.s008.docx]

**Table S6. Study 2: Separate Tobit Regressions of Donating on Social Class, Income, Education, and their Quadratic Terms (with Data from the American CEX)**

|  | ***N*** | ***Coeff.*** | ***t*** |
| --- | --- | --- | --- |
| Objective social class | 32,090 | .898 | 35.55*** |
| Objective social class² |  | -.118 | -5.41*** |
| Income | 32,090 | .624 | 25.81*** |
| Income² |  | .125 | 5.57*** |
| Educational status | 31,515 | .835 | 34.50*** |
| Educational status² |  | -.028 | -1.30 |

Predictor variables were standardized across all households.

*** *p* < .001 (two-tailed)
