# Supplementary material for: A Large Scale Test of the Effect of Social Class on Prosocial Behavior
Source: PLoS One. 2015 Jul 20;10(7):e0133193. doi: 10.1371/journal.pone.0133193 (PMC4507988; doi:10.1371/journal.pone.0133193)
Supplement: S7 Table — Predictor variables were standardized across all subjects separately for each year. Model 1 was computed including the covariates age and sex. Model 2 was computed without covariates. Sample sizes were different for each predictor variable (objective social class: N = 3,975; income: N = 3,536; educational status: N = 3,974; job prestige: N = 2,547). OR = odds ratio. b = estimated coefficient of the ordered probit model. a Logistic regresison (0 = nondonor; 1 = donor). b 0 = not at all in the past year; 5 = more than once a week. * p < .05. ** p < .01. *** p < .001 (two-tailed). (DOCX) [file pone.0133193.s009.docx]

**Table S7. Study 3: Separate Regressions of Donating on Social Class, Income, Education, Job Prestige, and their Quadratic Terms (with Data from the American GSS)**

|  | **Donating (yes/no)ª** | | **Frequency of donating^b^** | | | |
| --- | --- | --- | --- | --- | --- | --- |
|  |  |  | **Ordered probit model** | | **OLS regression model** | |
|  | ***OR*** | ***z*** | ***b*** | ***z*** | ***b*** | ***t*** |
| **Model 1**  **(including covariates)** |  |  |  |  |  |  |
| Objective social class | 2.54 | 18.29*** | .392 | 22.31*** | .447 | 23.04*** |
| Objective social class² | 0.95 | -1.15 | -.064 | -4.50*** | -.058 | -3.58*** |
| Income | 2.76 | 15.67*** | .440 | 19.74*** | .512 | 20.40*** |
| Income² | 1.19 | 4.71*** | .061 | 3.89*** | .084 | 4.80*** |
| Educational status | 2.32 | 16.08*** | .342 | 15.56*** | .401 | 15.79*** |
| Educational status² | 0.86 | -3.18** | -.077 | -4.18*** | -.081 | -3.75*** |
| Job prestige | 1.86 | 10.74*** | .269 | 12.72*** | .319 | 12.88*** |
| Job prestige² | 0.98 | -0.32 | -.048 | -2.62** | -.051 | -2.34* |
| **Model 2**  **(without covariates)** |  |  |  |  |  |  |
| Objective social class | 2.47 | 18.11*** | .379 | 21.76*** | .446 | 22.44*** |
| Objective social class² | 0.98 | -0.59 | -.054 | -3.81*** | -.048 | -2.93** |
| Income | 2.53 | 15.06*** | .411 | 18.66*** | .493 | 19.21*** |
| Income² | 1.16 | 4.00*** | .048 | 3.08** | .070 | 3.92*** |
| Educational status | 2.16 | 15.21*** | .302 | 13.93*** | .366 | 14.10*** |
| Educational status² | 0.93 | -1.59 | -.040 | -2.20* | -.039 | -1.78 |
| Job prestige | 1.91 | 11.26*** | .279 | 13.26*** | .338 | 13.44*** |
| Job prestige² | 0.98 | -0.44 | -.051 | -2.79** | -.055 | -2.50* |

Predictor variables were standardized across all subjects separately for each year. Model 1 was computed including the covariates age and sex. Model 2 was computed without covariates. Sample sizes were different for each predictor variable (objective social class: *N* = 3,975; income: *N* = 3,536; educational status: *N* = 3,974; job prestige: *N* = 2,547). *OR* = odds ratio. *b* = estimated coefficient of the ordered probit model.

*^a^* Logistic regresison (0 = nondonor; 1 = donor). ^b^ 0 = not at all in the past year; 5 = more than once a week.

* *p* < .05. ** *p* < .01. *** *p* < .001 (two-tailed).
