# Supplementary material for: A Large Scale Test of the Effect of Social Class on Prosocial Behavior
Source: PLoS One. 2015 Jul 20;10(7):e0133193. doi: 10.1371/journal.pone.0133193 (PMC4507988; doi:10.1371/journal.pone.0133193)
Supplement: S8 Table — Predictor variables were standardized per year across all subjects. Model 1 was computed including the covariates age and sex. Model 2 was computed without covariates. Sample sizes (observations) were different for each predictor variable (objective social class: N = 82,966; income: N = 74,053; educational status: N = 79,663; job prestige: N = 46,327). Observations were nested within persons. OR = odds ratio. b = estimated coefficient of the multilevel ordered probit model. a Multilevel logistic regression model (0 = nonvolunteer; 1 = volunteer). b 0 = never, 3 = every week. ** p < .01. *** p < .001 (two-tailed). (DOCX) [file pone.0133193.s010.docx]

**Table S8. Study 4: Separate Multilevel Regressions of Volunteering on Social Class, Income, Education, Job Prestige, and their Quadratic Terms (with Data from the German SOEP)**

|  | **Volunteering (yes/no)ª** | | **Frequency of volunteering^b^** | | | |
| --- | --- | --- | --- | --- | --- | --- |
|  |  |  | **Multilevel**  **ordered probit model** | | **Multilevel ordinary regression model** | |
|  | ***OR*** | ***z*** | ***b*** | ***z*** | ***b*** | ***z*** |
| **Model 1**  **(including covariates)** |  |  |  |  |  |  |
| Objective social class | 2.03 | 32.49*** | .336 | 29.10*** | .120 | 26.52*** |
| Objective social class² | 0.91 | -5.70*** | -.048 | -5.88*** | -.010 | -3.25** |
| Income | 1.46 | 19.62*** | .164 | 16.24*** | .060 | 14.90*** |
| Income² | 0.95 | -4.04*** | -.020 | -3.13** | -.005 | -1.99* |
| Educational status | 2.23 | 28.56*** | .407 | 26.19*** | .136 | 23.23*** |
| Educational status² | 0.86 | -5.73*** | -.093 | -6.33*** | -.021 | -3.72*** |
| Job prestige | 1.63 | 19.54*** | .213 | 16.20*** | .077 | 14.64*** |
| Job prestige² | 0.99 | -0.59 | -.008 | -0.84 | .001 | 0.23 |
| **Model 2**  **(without covariates)** |  |  |  |  |  |  |
| Objective social class | 2.03 | 32.51*** | .337 | 29.22*** | .122 | 27.09*** |
| Objective social class² | 0.92 | -5.57*** | -.047 | -5.74*** | -.010 | -3.11** |
| Income | 1.45 | 19.39*** | .163 | 16.16*** | .061 | 15.14*** |
| Income² | 0.95 | -3.81*** | -.019 | -2.98** | -.005 | -1.93 |
| Educational status | 2.28 | 29.38*** | .420 | 27.06*** | .142 | 24.33*** |
| Educational status² | 0.85 | -6.41*** | -.102 | -6.95*** | -.023 | -4.17*** |
| Job prestige | 1.66 | 20.28*** | .224 | 17.03*** | .082 | 15.56*** |
| Job prestige² | 1.00 | -0.09 | -.003 | -0.30 | .003 | 0.89 |

Predictor variables were standardized per year across all subjects. Model 1 was computed including the covariates age and sex. Model 2 was computed without covariates. Sample sizes (observations) were different for each predictor variable (objective social class: *N* = 82,966; income: *N* = 74,053; educational status: *N* = 79,663; job prestige: *N* = 46,327). Observations were nested within persons. *OR* = odds ratio. *b* = estimated coefficient of the multilevel ordered probit model.

*^a^* Multilevel logistic regression model (0 = nonvolunteer; 1 = volunteer). ^b^ 0 = never, 3 = every week.

** *p* < .01. *** *p* < .001 (two-tailed).
