# Supplementary material for: A Large Scale Test of the Effect of Social Class on Prosocial Behavior
Source: PLoS One. 2015 Jul 20;10(7):e0133193. doi: 10.1371/journal.pone.0133193 (PMC4507988; doi:10.1371/journal.pone.0133193)
Supplement: S9 Table — Predictor variables were standardized across all subjects separately for each year. Model 1 was computed including the covariates age and sex. Model 2 was computed without covariates. Sample sizes were different for each predictor variable (objective social class: N = 3,983; income: N = 3,540; educational status: N = 3,982; job prestige: N = 2,551). OR = odds ratio. b = estimated coefficient of the ordered probit model. a Logistic regression (0 = nondonor; 1 = donor). b 0 = not at all in the past year; 5 = more than once a week. * p < .05. ** p < .01. *** p < .001 (two-tailed). (DOCX) [file pone.0133193.s011.docx]

**Table S9. Study 5: Separate Regressions of Volunteering on Social Class, Income, Education, Job Prestige, and their Quadratic Terms (with Data from the American GSS)**

|  | **Volunteering (yes/no)ª** | | **Frequency of volunteering^b^** | | | |
| --- | --- | --- | --- | --- | --- | --- |
|  |  |  | **Ordered probit model** | | **OLS regression model** | |
|  | ***OR*** | ***z*** | ***b*** | ***z*** | ***b*** | ***t*** |
| **Model 1**  **(including covariates)** |  |  |  |  |  |  |
| Objective social class | 1.64 | 14.50*** | .248 | 13.49*** | .291 | 12.86*** |
| Objective social class² | 1.02 | 0.53 | .017 | 1.15 | .047 | 2.50* |
| Income | 1.50 | 9.57*** | .201 | 8.69*** | .240 | 8.02*** |
| Income² | 1.10 | 3.11** | .054 | 3.28** | .073 | 3.49*** |
| Educational status | 1.77 | 12.97*** | .294 | 12.18*** | .333 | 11.49*** |
| Educational status² | 0.89 | -3.28** | -.051 | -2.55* | -.026 | -1.04 |
| Job prestige | 1.49 | 9.56*** | .219 | 9.66*** | .263 | 9.31*** |
| Job prestige² | 1.02 | 0.48 | .004 | 0.23 | .022 | 0.89 |
| **Model 2**  **(without covariates)** |  |  |  |  |  |  |
| Objective social class | 1.62 | 14.27*** | .244 | 13.27*** | .287 | 12.63*** |
| Objective social class² | 1.01 | 0.36 | .016 | 1.09 | .048 | 2.52* |
| Income | 1.47 | 9.20*** | .187 | 8.16*** | .223 | 7.47*** |
| Income² | 1.10 | 3.10** | .050 | 3.07** | .068 | 3.26** |
| Educational status | 1.79 | 13.25*** | .294 | 12.22*** | .329 | 11.40*** |
| Educational status² | 0.87 | -3.84*** | -.053 | -2.70** | -.025 | -1.01 |
| Job prestige | 1.47 | 9.47*** | .221 | 9.79*** | .268 | 9.51*** |
| Job prestige² | 1.01 | 0.31 | .000 | -0.03 | .015 | 0.62 |

Predictor variables were standardized across all subjects separately for each year. Model 1 was computed including the covariates age and sex. Model 2 was computed without covariates. Sample sizes were different for each predictor variable (objective social class: *N* = 3,983; income: *N* = 3,540; educational status: *N* = 3,982; job prestige: *N* = 2,551). *OR* = odds ratio. *b* = estimated coefficient of the ordered probit model.

*^a^* Logistic regresison (0 = nondonor; 1 = donor). ^b^ 0 = not at all in the past year; 5 = more than once a week.

* *p* < .05. ** *p* < .01. *** *p* < .001 (two-tailed).
