# Supplementary material for: A Large Scale Test of the Effect of Social Class on Prosocial Behavior
Source: PLoS One. 2015 Jul 20;10(7):e0133193. doi: 10.1371/journal.pone.0133193 (PMC4507988; doi:10.1371/journal.pone.0133193)
Supplement: S10 Table — Predictor variables were standardized across all subjects separately for each country. Model 1 was computed including the covariates age and sex. Model 2 was computed without covariates. Sample sizes were different for each predictor variable (objective social class: N = 37,136; income: N = 25,622; educational status: N = 36,695; job prestige: N = 22,764). Subjects were nested within countries. OR = Odds Ratio. b = estimated coefficient of the multilevel ordered probit model. a Multilevel logistic model (0 = nonvolunteer; 1 = volunteer). b 0 = no; 3 = yes, 6 or more times. ** p < .01. *** p < .001 (two-tailed). (DOCX) [file pone.0133193.s012.docx]

**Table S10. Study 6: Separate Multilevel Regressions of Volunteering on Social Class, Income, Education, Job Prestige, and their Quadratic Terms (with Data from the ISSP)**

|  | **Volunteering (yes/no)ª** | | **Frequency of volunteering^b^** | | | |
| --- | --- | --- | --- | --- | --- | --- |
|  |  |  | **Multilevel**  **ordered probit model** | | **Multilevel ordinary regression model** | |
|  | ***OR*** | ***z*** | ***b*** | ***z*** | ***b*** | ***z*** |
| **Model 1**  **(including covariates)** |  |  |  |  |  |  |
| Objective social class | 1.18 | 6.46*** | .094 | 6.60*** | .058 | 6.56*** |
| Objective social class² | 0.97 | -2.50* | -.012 | -2.14* | -.004 | -1.05 |
| Income | 0.98 | -1.18 | -.017 | -1.38 | -.012 | -1.41 |
| Income² | 1.00 | 0.28 | .003 | 0.51 | .003 | 0.75 |
| Educational status | 1.24 | 7.14*** | .125 | 7.10*** | .081 | 7.00*** |
| Educational status² | 0.98 | -1.27 | -.007 | -0.86 | -.001 | -0.11 |
| Job prestige | 1.11 | 4.22*** | .063 | 4.53*** | .044 | 4.73*** |
| Job prestige² | 1.02 | 2.05* | .012 | 1.75 | .009 | 1.70 |
| **Model 2**  **(without covariates)** |  |  |  |  |  |  |
| Objective social class | 1.16 | 6.13*** | .084 | 6.29*** | .051 | 6.06*** |
| Objective social class² | 0.98 | -1.62 | -.005 | -0.94 | .001 | 0.26 |
| Income | 1.02 | 1.02 | / | / | .013 | 1.58 |
| Income² | 0.99 | -0.73 | / | / | -.003 | -0.78 |
| Educational status | 1.17 | 5.48*** | .084 | 5.19*** | .051 | 4.62*** |
| Educational status² | 1.02 | 1.26 | .016 | 1.91 | .016 | 2.62** |
| Job prestige | 1.11 | 4.41*** | .065 | 4.70*** | .046 | 4.79*** |
| Job prestige² | 1.03 | 2.26* | .014 | 2.06* | .010 | 2.08* |

Predictor variables were standardized across all subjects separately for each country. Model 1 was computed including the covariates age and sex. Model 2 was computed without covariates. Sample sizes were different for each predictor variable (objective social class: *N* = 37,136; income: *N* = 25,622; educational status: *N* = 36,695; job prestige: *N* = 22,764). Subjects were nested within countries. *OR* = Odds Ratio. *b* = estimated coefficient of the multilevel ordered probit model.

*^a^* Multilevel logistic model (0 = nonvolunteer; 1 = volunteer). ^b^ 0 = no; 3 = yes, 6 or more times.

* *p* < .05. ** *p* < .01. *** *p* < .001 (two-tailed).
