# Supplementary material for: A Large Scale Test of the Effect of Social Class on Prosocial Behavior
Source: PLoS One. 2015 Jul 20;10(7):e0133193. doi: 10.1371/journal.pone.0133193 (PMC4507988; doi:10.1371/journal.pone.0133193)
Supplement: S11 Table — Predictor variables were standardized across all subjects separately for each year. Model 1 was computed including the covariates age and sex. Model 2 was computed without covariates. Sample sizes were different for each predictor variable (objective social class: N = 3,902; income: N = 3,486; educational status: N = 3,901; job prestige: N = 2,496). b = unstandardized regression coefficients. * p < .05. ** p < .01. *** p < .001 (two-tailed). (DOCX) [file pone.0133193.s013.docx]

**Table S11. Study 7: Separate Regressions of Everyday Helping on Social Class, Income, Education, Job Prestige, and their Quadratic Terms (with Data from the American GSS)**

|  | ***b*** | ***t*** |
| --- | --- | --- |
| **Model 1 (including covariates)** |  |  |
| Objective social class | .397 | 4.74*** |
| Objective social class² | -.110 | -1.58 |
| Income | .303 | 2.80** |
| Income² | .181 | 2.40* |
| Educational status | .567 | 5.30*** |
| Educational status² | -.236 | -2.60** |
| Job prestige | .407 | 3.95*** |
| Job prestige² | -.161 | -1.78 |
| **Model 2 (without covariates)** |  |  |
| Objective social class | .406 | 4.74*** |
| Objective social class² | -.147 | -2.06* |
| Income | .385 | 3.52*** |
| Income² | .228 | 2.95** |
| Educational status | .678 | 6.23*** |
| Educational status² | -.366 | -3.98*** |
| Job prestige | .312 | 2.98** |
| Job prestige² | -.123 | -1.34 |

Predictor variables were standardized across all subjects separately for each year. Model 1 was computed including the covariates age and sex. Model 2 was computed without covariates. Sample sizes were different for each predictor variable (objective social class: *N* = 3,902; income: *N* = 3,486; educational status: *N* = 3,901; job prestige: *N* = 2,496). *b* = unstandardized regression coefficients.

* *p* < .05. ** *p* < .01. *** *p* < .001 (two-tailed).
