# Supplementary material for: A Large Scale Test of the Effect of Social Class on Prosocial Behavior
Source: PLoS One. 2015 Jul 20;10(7):e0133193. doi: 10.1371/journal.pone.0133193 (PMC4507988; doi:10.1371/journal.pone.0133193)
Supplement: S12 Table — Predictor variables were standardized per year across all subjects. Model 1 was computed including the covariates age and sex. Model 2 was computed without covariates. Sample sizes (observations) were different for each predictor variable and player (objective social class: N = 1,901/1,918; income: N = 1,785/1,809; educational status: N = 1,842/1,881; job prestige: N = 946/1,031). Observations were nested within persons. b = unstandardized regression coefficients. * p < .05. *** p < .001 (two-tailed). (DOCX) [file pone.0133193.s014.docx]

**Table S12. Study 8: Separate Multilevel Generalized Linear Models for Testing the Effects of Social Class, Income, Education, Job Prestige, and their Quadratic Terms on Points Sent in the Trust Game (with Data from the German SOEP)**

|  | **Player 1** | | **Player 2** | |
| --- | --- | --- | --- | --- |
|  | ***b*** | ***z*** | ***b*** | ***z*** |
| **Model 1 (including covariates)** |  |  |  |  |
| Objective social class | .468 | 5.49*** | .421 | 5.54*** |
| Objective social class | -.024 | -0.35 | -.075 | -1.19 |
| Income | .350 | 4.60*** | .293 | 4.20*** |
| Income² | .009 | 0.17 | -.020 | -0.40 |
| Educational status | .262 | 2.38* | .468 | 5.35*** |
| Educational status² | .058 | 0.58 | -.142 | -1.83 |
| Job prestige | .540 | 4.94*** | .221 | 2.35* |
| Job prestige² | -.020 | -0.22 | .034 | 0.43 |
| **Model 2 (without covariates)** |  |  |  |  |
| Objective social class | .443 | 5.18*** | .403 | 5.31*** |
| Objective social class² | -.026 | -0.37 | -.073 | -1.15 |
| Income | .329 | 4.31*** | .294 | 4.20*** |
| Income² | .010 | 0.19 | -.020 | -0.41 |
| Educational status | .273 | 2.47* | .431 | 4.95*** |
| Educational status² | .039 | 0.39 | -.139 | -1.78 |
| Job prestige | .539 | 4.94*** | .207 | 2.21* |
| Job prestige² | -.019 | -0.21 | .041 | 0.52 |

Predictor variables were standardized per year across all subjects. Model 1 was computed including the covariates age and sex. Model 2 was computed without covariates. Sample sizes (observations) were different for each predictor variable and player (objective social class: *N* = 1,901/1,918; income: *N* = 1,785/1,809; educational status: *N* = 1,842/1,881; job prestige: *N* = 946/1,031). Observations were nested within persons. *b* = unstandardized regression coefficients.

* *p* < .05. *** *p* < .001 (two-tailed).
